# Interspecific Hybrids Between *Pelargonium* × *hortorum* and Species From *P.* Section *Ciconium* Reveal Biparental Plastid Inheritance and Multi-Locus Cyto-Nuclear Incompatibility

**DOI:** 10.3389/fpls.2020.614871

**Published:** 2020-12-18

**Authors:** Floris C. Breman, Ronald C. Snijder, Joost W. Korver, Sieme Pelzer, Mireia Sancho-Such, M. Eric Schranz, Freek T. Bakker

**Affiliations:** ^1^Biosystematics Group, Wageningen University and Research, Wageningen, Netherlands; ^2^Syngenta Seeds BV, Andijk, Netherlands

**Keywords:** *Pelargonium*, cyto-nuclear incompatibility, interspecific hybridization, biparental inheritance, plastid

## Abstract

The genetics underlying Cyto-Nuclear Incompatibility (CNI) was studied in *Pelargonium* interspecific hybrids. We created hybrids of 12 closely related crop wild relatives (CWR) with the ornamental *P.* × *hortorum*. Ten of the resulting 12 (F_1_) interspecific hybrids segregate for chlorosis suggesting biparental plastid inheritance. The segregation ratios of the interspecific F_2_ populations show nuclear interactions of one, two, or three nuclear genes regulating plastid function dependent on the parents. We further validated that biparental inheritance of plastids is common in section *Ciconium*, using diagnostic PCR primers. Our results pave the way for using the diverse species from section *Ciconium*, each with its own set of characteristics, as novel sources of desired breeding traits for *P.* × *hortorum* cultivars.

## Introduction

Several closely related species from *Pelargonium* sect. *Ciconium* have been used for producing hybrids that are sold world-wide, commonly known as “garden geraniums,” that are some of the most popular and iconic ornamentals. However, there are genetic barriers to establishing crosses and making new hybrids, including Cyto-Nuclear Incompatibility (CNI) that can cause cytoplasmic male sterility (CMS), dwarf growth (DG) and chlorosis in hybrid offspring (Greiner et al., 2015; Postel and Touzet, 2020). Nearly all angiosperms have uniparental maternal organelle inheritance. Unusually, *Pelargonium* × *hortorum* as well as the species *P. zonale* display biparental inheritance of their organelles (Baur, 1909; Tilney-Bassett et al., 1992; Weihe et al., 2009). Inheritance of organelles in plants with biparental transmission was found to be non-Mendelian in these studies, even though the expression of organelles is managed by the nuclear genome (Barkan and Small, 2014; Börner et al., 2015; Zhang and Lu, 2019). Phenotypic effects of plastid types in otherwise equal nuclear genomic backgrounds were recently demonstrated in *Arabidopsis* (Flood et al., 2020), but other such studies are so far rare.

*Pelargonium* species are an attractive model system to study CNI as different organellar effects can be evaluated in offspring with equal nuclear-genomic backgrounds, using established crossing techniques. There is a long history of observations of CNI in *Pelargonium* starting in the twentieth century when the foundations were laid for some of the cultivars we have today (e.g., Sweet 1820, 1822). Subsequently, more detailed studies of CNI, especially plastid-induced, were carried out in *Pelargonium* (Baur, 1909; Tilney-Bassett, 1973, 1974, 1975), which, based on segregation ratios, ultimately found support for a two-gene model of complementary nuclear genomic alleles that control the inheritance of organelles in *Pelargonium* (Tilney-Bassett, 1976, 1984, 1988; Tilney-Bassett and Birky, 1981; Tilney-Bassett and Abdel-Wahab, 1982; Tilney-Bassett et al., 1989b).

To further advance our knowledge of CNI in *Pelargonium*, we have performed a section-wide survey of most of the crop wild relatives (CWR) of *P.* × *hortorum* and its supposed ancestors *P. inquinans* and *P. zonale* (James et al., 2004) to investigate the inheritance of organelles in general and plastids in particular. There are currently 17 species recognized in *Pelargonium* section *Ciconium* (van der Walt and Vorster, 1988; Röschenbleck et al., 2014) which are all considered the CWR of *P × hortorum*. Phylogenetic relationships among these species have recently been reconstructed based on 76 plastome exon sequences (van de Kerke et al., 2019 and references therein). We further investigated if chlorosis in the hybrid offspring can be correlated with a particular plastid type (e.g., the combined plastid proteome, metabolome, and transcriptome inherited from one parent). Given the ubiquitous occurrence of chlorosis in crosses between species of *P.* sect. *Ciconium* and in other sections in the genus (Sweet 1820–1822, Horn, 1994; Breman pers. obs.), we expect that biparental inheritance of organelles is more common than is currently reported in the published literature.

Finally, based on segregation ratios over one of the crossing series, we deduced the underlying model of interacting genes which can explain the occurrence of chlorotic phenotypes in these crosses, and hence CNI. We did this by disentangling the effects of each possible plastome type on chlorosis in the F1 species hybrids.

## Materials and Methods

**FIGURE 1:**
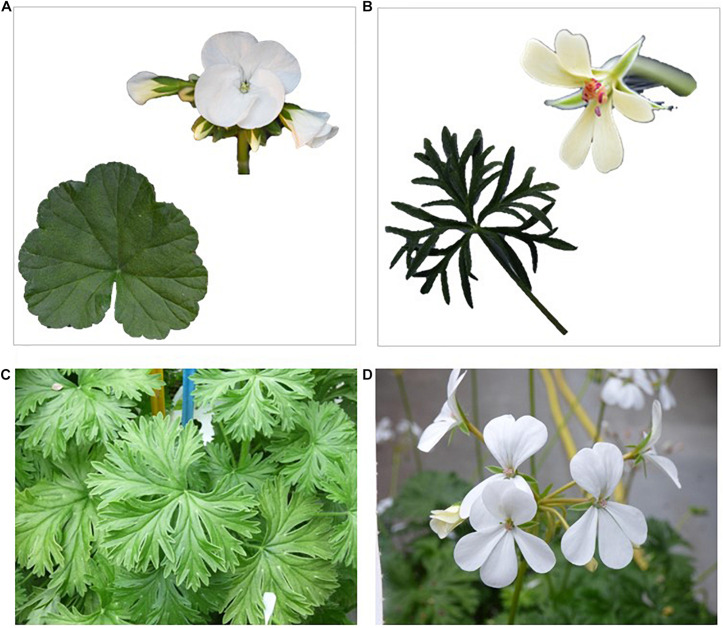
Example of the phenotype of an F1 hybrid generated from the HORT × ARID cross. **(A)** Shows the leaf and flower phenotypes of *P.* × *hortorum*; **(B)** shows the leaf and flower phenotypes of *P. aridum*; **(C)** shows the leaf phenotype of F_1_ HORT × ARID; **(D)** shows the flower phenotype of F1 HORT × ARID.

We established novel interspecific crossings between twelve related diploid species of *P.* section *Ciconium* and *P. × hortorum* (species and acronyms mentioned in Table 1). We verified the hybrid status of the offspring using phenotyping, especially by evaluating leaf morphology, as well as flower color and shape (for an example see Figure 1 for all others see Supplementary Figure 4). In addition, hybrid status and ploidy level of obtained F_1_ hybrids were verified by flow-cytometry using *P.* × *hortorum* as internal reference. Flow-cytometry was performed by Iribov bv (Heerhugowaard, Netherlands) on freshly collected leaf material using a Partec CA-II flowcytometer according to De Laat et al. (1987). Nuclei were stained with a High-Resolution Kit (Partec).

**TABLE 1:** Plant materials used in this study.

Species	Herbarium voucher	Species acronym used in the text	Institute^*a*^
*P. acetosum*	1243	ACET	NHM
*P. acraeum*	1975	ACRA	STEU
*P. alchemilloides*	1885	ALCH	STEU
*P. articulatum*	S1026	ARTI	SYN
*P. barklyi*	S1027	BARK	SYN
*P. frutetorum*	S1087	FRUT	SYN
*P. inquinans*	0682	INQU	STEU
*P. multibracteatum*	2902	MULT	STEU
*P. peltatum*	1890	PELT	STEU
*P. quinquelobatum*	S1044	QUIN	SYN
*P. ranunculophyllum*	A3651	RANU	MSUN(*)
*P. tongaense*	3074	TONG	STEU
*P. zonale*	1896	ZONA	STEU
*P. elongatum*	0854	ELON	STEU
*P. aridum*	S1088	ARID	SYN
*P. × hortorum* “Pinto white” (PW)	PEZ-BD8517	HORT	SYN
*P. × hortorum* “Tango White” (TW)	NA	HORT	NA

*Herbarium voucher information. ^a^STEU, Stellenbosch University, RSA; AL, Albers/MSUN, Münster Germany; SYN, Syngenta collection number; NHM, Natural History Museum London UK.*

### Interspecific F_1_ Hybrids

**FIGURE 2:**
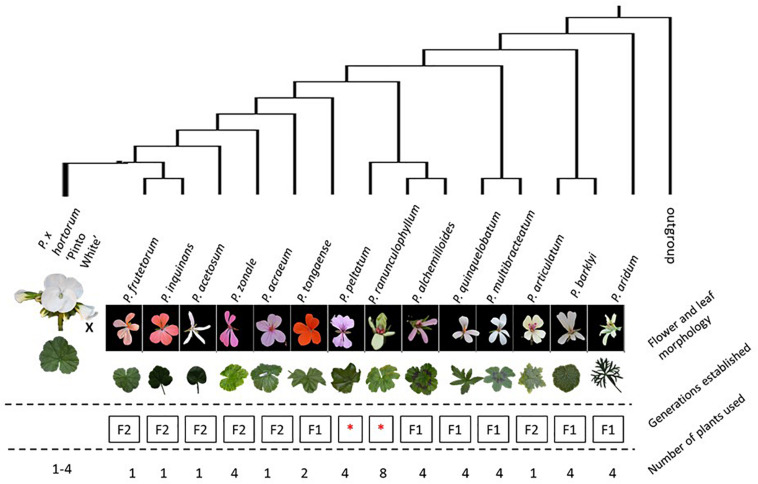
Schematic overview of the experimental setup for the F_1_ crosses put in a phylogenetic context. The tree displayed tree is based on van de Kerke et al. (2019). The position of *P. ranunculophyllum* is inferred from our plastome assembly data (Breman et al. in prep), combined with those of van de Kerke et al. (2019) and Breman et al. (in prep). On the top row are all accessions used, represented by their respective floral and leaf phenotypes and structured by their presumed phylogenetic distance to “HORT” (van de Kerke et al., 2019); “Generations established” indicates results obtained with “F_1_” indicating only F_1_ plants were obtained, no F_2_ could be generated; “F_2_” meaning the plants were fertile and could produce F_2_ offspring; red asterisks denote crosses that failed for reasons explained in the text. The bottom row indicates the number of plants used across the seasons.

The F_1_ hybrids generated in this study were produced from the diploid HORT cultivar “Pinto White” (PW) crossed with the species outlined above (listed in Table 1 and Figure 2). In addition, we attempted one interspecific cross at the tetraploid level using HORT “Tango White” (TW) and *P. articulatum* (ARTI). For all crosses, plants were moved to a pre-cleaned greenhouse and manually pollinated by using dedicated small paint brushes, made of animal hair, at 1-day intervals from the moment of flowering, dependent on the species. When seed development did not take place or was impaired, embryo rescue (Table 2) was performed as follows: at 2–3 weeks after pollination, embryos were collected, dissected and put on tissue culture in dedicated cabinets using an approach similar to Kamlah et al. (2019).

**TABLE 2:** F_1_ offspring overview.

Hybrid	Origins of plastid	Phenotype	# offspring obtained
HORT × ZONA	Maternal	Chlorotic	144
HORT × ZONA	Paternal	Mostly Green	
HORT × ZONA	Biparental	Variegated	
HORT × ACET	Paternal	Green Vir	7
HORT × ACET	Maternal	Lethal	
HORT × FRUT	NP	Green	72
HORT × INQU	NP	Green	2
HORT × ACRA	Paternal	Green	24
HORT × QUIN	Maternal	Lethal	12
HORT × QUIN	Paternal	Chlorotic Vir	
HORT × QUIN	Biparental	Variegated	
HORT × MULT	Paternal	Chlorotic Vir	21
HORT × ALCH	Paternal	Chlorotic Vir	8
HORT × TONG	Paternal	Chlorotic	36
HORT × ARID	Paternal	Chlorotic	10
HORT × ARID	Maternal	Lethal	
HORT × PELT	*	–	–
HORT × RANU	*	–	–
HORT × BARK	Paternal	Lethal	2
HORT^4^ × ARTI	Either	Green	–
HORT^4^ × ARTI	Eihter	Chlorotic	
HORT^4^ × ARTI	Either	Lethal	

Vir indicates plants were virescent. *Crosses failed either because (paternal) plants would not flower or no fruit was ever observed. **HORT^4^** refers to a tetraploid cultivar from HORT called “Tango White” All other HORT refer to the diploid “Pinto White” cultivar.

### Interspecific F_2_ Populations

**FIGURE 3:**
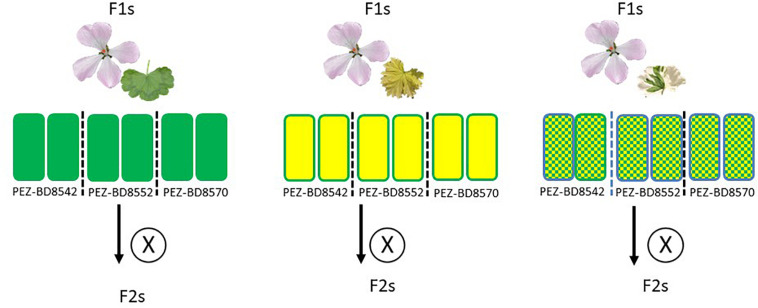
Experimental setup for obtaining F_2_ crosses of F_1_ parents with different phenotypes.

In order to evaluate the nuclear background of CNI, we created F_2_ progeny of particular F_1_ individuals (Table 3). We selected F_1_ plants which we assume to contain either one, or both parental cytotypes based on overall leaf coloration. We hypothesized that green and chlorotic plants contained one parental type (at that point unknown which one) and that variegated plants contained both (biparental). We selected from these a number of individuals for subsequent self-pollination to generate the F_2_ populations: six plants in total representing three phenotypes encountered in the HORT × ZONA F_1_ which includes 2 green (denoted as: HORT × ZONA^*G*^’), 2 variegated (denoted as: “HORT × ZONA^*V*^”) and 2 chlorotic (denoted as: “HORT × ZONA^*C*^”) plants (see Figures 3, 5). In addition, we included one cross (three green plants, the only surviving phenotype) involving *P. acetosum* (*ACET*). We also selected plants from a crossing involving *P. frutetorum* (*FRUT*) and *P. inquinans* (*INQU*) as positive controls for the evaluation. This is because *Pinto White* contains a plastid that is considered to have originated from the *P. inquinans* ancestor (James et al., 2004) and the plastid of *P. frutetorum* is indistinguishable from that of *PW* and *P. inquinans* (Breman et al., in prep). Therefore, we expected these crosses not to display chlorosis in the F_2_.

**TABLE 3:** Genotypes detected in F1 and F2 offspring, using diagnostic PCR, for the HORT × ZONA cross.

Pedigree	Plant/cross	Phenotype	Origin of plastid
F0	Hortorum	G	Wild-type M
	Zonale	G	Wild-type P
F1	8542	G	P
	8542	C	M
	8552	G	P
	8552	V	M
	8570	C	M
	8570	G	P
	8570	C	P
	8570	V	M
PEZ-BD8542	8618	C	G-P
	8618	G	G-P
	8618	C	NA
	8619	G	G-P
	8619	C	G-P
	8620	C	G-M
	8620	C	G-M
	8620	C	G-M
	8620	C	G-M
	8627	C	G-P
	8627	G	G-P
	8628	C	G-M
	8628	V	biparental
	8628	G	G-P
	8628	C	G-M
	8629	C	G-M
PEZ-BD85552	8621	C	NA
	8621	G	G-P
	8623	C	G-M
	8623	G	G-M
	8630	C	G-P
	8630	G	G-P
	8631	C	G-M
	8631	G	G-M
	8632	Lethal	G-M
	8632	C	G-M
PEZ-BD8570	8624	C	G-M
	8625	C	G-M
	8625	C	G-M
	8625	G	G-M
	8626	G	G-P
	8626	C	G-P
	8634	Lethal	G-M
	8634	G	G-P
	8634	C	G-M

*Structured by cross. (G)-P and (G)-M denotes (grand) paternally-and maternally inherited plastids.*

We also evaluated a subset of plants for evaluation of segregation for CNI phenotype patterns that are expressed during the pre-seedling phase. We selected three F_1_ parents of HORT × ACET^*G*^, four HORT × ZONA^*G*^ and one parent each of HORT × FRUT^*G*^ and HORT × ACRA^*G*^. Because fruit-set was low this season for HORT × ZONA we pooled these to enable Chi^2^ testing. We feel pooling was justified, because these plants share parentage, and have the same phenotype and plastid.

#### Plant Rearing

Plants were grown in a greenhouse from seeds and leaf material was collected from the first primary leaves for DNA extraction. See Table 1 for the full list of plant material used with Herbarium accession numbers and see Supplementary Figures 6A–K for representative phenotypes of each F1 plant.

#### DNA Extraction, Primer Design, and Genotyping

Genomic DNA was extracted from leaf material using a modified CTAB protocol (Bakker et al., 1998) followed by RNAse treatment. We designed specific primers for plastome-typing parents and F1 offspring We used the Long Single Copy region (LSC) of assembled plastomes (Breman et al., in prep) for *Pelargonium* section *Ciconium* species. LSC has been shown to contain numerous indels (Chumley et al., 2006; Guisinger et al., 2008, 2011; Weng et al., 2017; Breman et al., in prep) which can be used to create genotype-specific primer sites. Visual inspection of sequence alignments, combined with parsimony analysis and using the “Apomorphy list” command in PAUP^∗^4b10 for windows (Swofford, 2002), was performed to find suitable primer sites and to check for unique autapomorphies therein. We specifically scanned for regions with a unique indel or multiple unique substitutions, allowing for genotype-specific primers. Amplicon sizes were designed to be < 500 bp, allowing for shorter PCR thermo-profiles. Candidate primer pairs were evaluated using Oligocalc (Kibbe, 2007)^1^ checking for differences between melting temperatures (ΔTm), self-priming and hairpin formation. Primers were accepted when ΔTm between forward and reverse primers was < 3°C and with only one hairpin and/or one self-priming was predicted. Further, we required a primer site to have a minimum Illumina read coverage of 20. A GC content of 40–50% was preferred, but this was not always possible. A GC content of 40–50% is considered best for ensuring stable binding during annealing and increase the primer pairs efficiency. Finally, we submitted the primers to a BLAST search (set for analyzing short sequences) to compare to all available *Pelargonium* sequences to verify target-specificity. Occasionally a single primer would have a significant hit to *Pelargonium* species outside section *Ciconium*, but this never occurred for both primers of a pair.

Primers were tested *in vitro*, using a panel of 16 section *Ciconium* species representing the range of parental plastid variation we would encounter in our offspring. Primer candidates were evaluated using the target accession and an annealing temperature gradient ranging from 49 to 60°C. Primers that amplified were subsequently tested against the panel of accessions at the highest possible temperature for which it showed amplification of the target. For PCR profiles and reaction conditions see Supplementary Figure 3.

#### Phenotyping of F_1_ and F_2_ Plants

**FIGURE 4:**
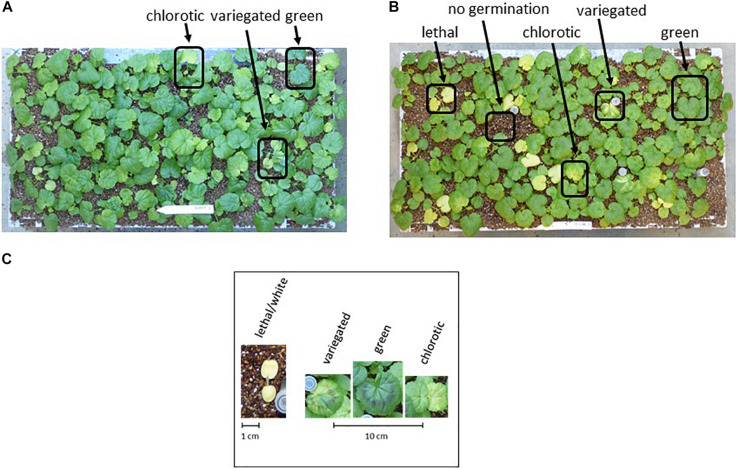
Chlorosis phenotypes. **(A)** Segregation for chlorosis among progeny in the F_2_ HORT × FRUT. **(B)** Segregation for chlorosis in F_1_ HORT × ZONA. Examples of “green,” “chlorotic” and “variegated” plants are indicated, as well as “lethal” phenotypes and seeds that failed to germinate. **(C)** Scaled close-up of examples of phenotypes.

**FIGURE 5:**
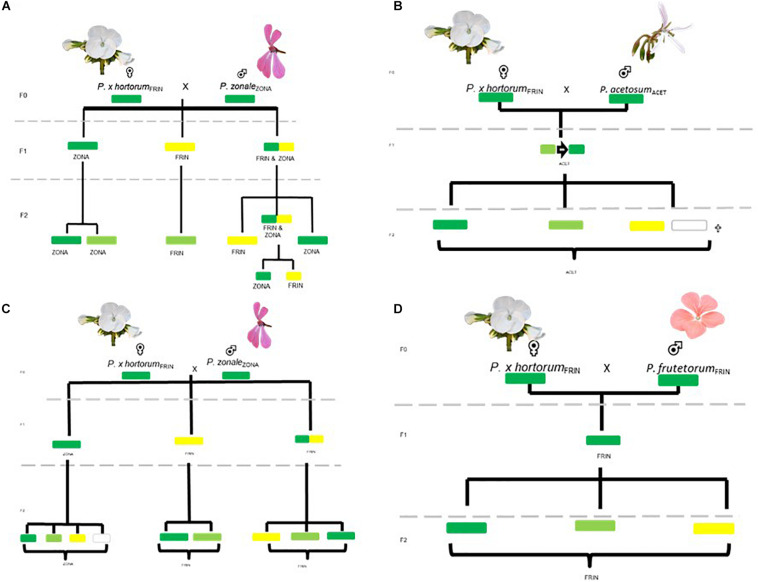
Tracing plastids throughout the pedigrees. Correlation between phenotype and genotype, subsequent segregation in F_2_ offspring is also indicated. **(A,B)** TWO HORT × ZONA crosses; **(C)** the HORT × ACET cross; **(D)** the HORT × FRUT cross. Text in subscript denotes the plastid type found in the parental population.

Leaf color phenotyping was performed at the seedling stage (Figures 4A,B). In order to consistently compare phenotypes across populations per cross, we took photos of seedlings at 2-week intervals during the seedling stage until the development of the first two primary leaves (Figure 4). We used the following four leaf-phenotyping categories based on a visual assessment of the phenotypes: (1) “Green”: leaf phenotype comparable to parents; (2) “Chlorotic,” plants are lighter green than either parent or even yellow; (3) “White,” plants germinate, but die within 2 weeks. Seeds that failed to germinate are added to this category; (4) “Variegated,” plants display more than one chlorotic phenotype in the same individual, presumably due to heteroplasmy (see Figures 4B,C, 5).

Ratios of the four phenotypes for each tested F_2_ population were compared and fitted to a one-, two- and three-gene model of inheritance of nuclear genomic alleles (calculated using the spreadsheet from Montoliu, 2012). We assumed four phenotypes and combined these according to five different scenarios, each representing assumptions on expected phenotypic ratios and their expression. The first scenario tested considers four phenotypes (i.e., “not affected,” “mildly,” “severely,” and “lethal”). The second and third scenarios consider there to be three phenotypes (“not affected,” “affected,” and “lethally affected”). Finally, the fourth and fifth scenarios consider only two phenotypes (“affected” vs. “not affected”). We then evaluated these five different scenarios by binning individuals differently. E.g., under scenarios two and three only green plants are considered to be unaffected but the lethal category consisted either of only the white or the white and severely affected plants (Table 4A). Thereby we further assumed different parental genotypes and their expected phenotypic ratios leading to eight testable phenotypic ratios representing models of one, two, or three loci involved (Table 4B).

**TABLE 4A:** Crosses matching genetic models of inheritance.

		One gene model			Two gene model			Three gene model		Observed ratios
Scenarios	Crosses meeting criteria	a	b	c	d	e	f	g	h	
Scenario 1	F2_hort_x_zona^*V*^	M***	–	–	–	–	M***	–	M***	∼4:5:1:3
Scenario 2	F2_hort_x_zona^*G*^	–	–	M***	–	–	–	–	–	∼7:7:1
	F2_hort_x_zona^*V*^	M***	M***	–	–	–	M***	–	M***	∼1:2:1
	F2_hort_x_frut	–	M***	–	–	–	M***	–	M***	∼1:3:0
Scenario 3	None	–	–	–	–	–	–	–	–	–
Scenario 4	None	–	–	–	–	–	–	–	–	–
Scenario 5	F2_hort_x_zona^*G*^	–	–	M***	–	–	–	–	–	∼1:1!^50:50^
	F2_hort_x_zona^*V*^	M***	M***	–	–	–	M***	–	M***	∼1:2!^75:25^
	F2_hort_x_frut	–	M***	–	–	–	M***	–	M***	∼1:3!^75:25^
Fruit/seed phase										
Scenario 1	None	–	–	–	–	–	–	–	–	–
Scenario 2	None	–	–	–	–	–	–	–	–	–
Scenario 3	None	–	–	–	–	–	–	–	–	–
Scenario 4	F2_hort_x_frut	–	–	M***	–	–	–	–	–	1:1!**^50:50^**
	F2_hort_x_zona^*G*^	—-	M***	–	–	–	M***	–	M***	∼1:3**!^25:75^**
	F2_hort_x_acet	–	M***		–	–	M***	–	M***	∼1:3!**^25:75^**
Scenario 5	F2_hort_x_frut	–	M***		–	–	M***	–	–	∼1:4
	F2_hort_x_acra	–	–	M***	–	–	–	–	–	∼1:1.5
	F2_hort_x_zona^*G*^	–	M***	–	–	–	M***	–	–	∼1:1.3

M: mendelian model applies, ***P < 0.001 under the χ^2^-test! indicates ratios matching particular model. For all observed ratios and counts of phenotypes under each scenario please see Supplementary Material 5.

**TABLE 4B:** Possible parental genotype and expected phenotypic ratios.

Lettercode	Genetic model and expected ratios
a	F1xF1 = AaxAa 25:50:25
b	F1xF1 = AaxAa 25:75
c	F1xF1 = AaxAA 50:50
d	F1xF1 = AaBbxAaBb 6.25:18.75:18.75:56.25
e	F1xF1 = AABbxAaBB 25:25:25:25
f	F1xF1 = AABbxAaBB 25:75
g	F1xF1 = AaBbCcxAaBbCC 6.25:18.75:56.25:18.75
h	F1xF1 = AaBbCcxAaBbCC 25:75

**FIGURE 6:**
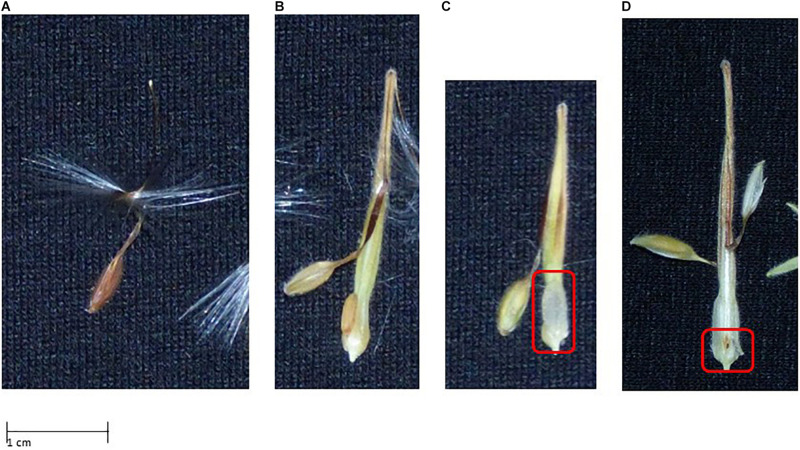
Seed phenotypes displaying signs of Cyto-nuclear incompatibility (see text for further explanation). **(A)** Normal seed phenotype; **(B)** bleached seed phenotype; **(C)** seed containing no or watery endosperm; **(D)** no embryo development.

For evaluating seed phenotypes, we used a similar approach, distinguishing four phenotypes: (1) “normal,” not affected by CNI, 2); “bleached,” seed contains endosperm that is still filled, but the seed is bleached; (3) “watery,” in this case the endosperm is bleached and not properly filled; (4) “lethal,” seeds with this phenotype displayed early aborted or undeveloped embryos. For examples see Figure 6.

As for leaf phenotypes, for seed phenotyping we evaluated five scenarios as well. We assumed four (“normal,” “bleached,” “empty,” “lethal,” scenario 1), three (“not affected,” “affected,” and “lethally affected,” scenarios 2 and 3) and two (“affected” vs. “not affected,” scenarios 4 and 5) phenotypes.

#### Genotyping F_1_ Plants and F_2_ Populations

We plastome-typed F_1_ plants using our diagnostic primers described above (Table 3). In those cases where the F_1_ population segregated for chlorosis, we tested accessions representing each phenotype. We then typed F_2_ plants from each population, and plastome types were then associated with the measured leaf phenotypes to establish the correlation, and thus effect, of each plastid type in the segregating offspring.

## Results

### Crossing Results

#### Interspecific F_1_ Hybrids

From thousands of pollination attempts we created a total of 314 F_1_ hybrid plants from crossing our species panel to the ornamental cultivar *P. × hortorum* PW (see Table 2). Twelve interspecific crosses were successful in producing F_1_ plants (Table 2). For three crosses embryo rescue (ER) was needed In order to produce scorable progeny, whereas three attempted crossings failed. Attempts to cross HORT with *P. elongatum* (ELON) failed, but this was expected given the difference in basic chromosome numbers between the two accessions (HORT × = 9, *P. elongatum* × = 4 (Gibby and Westfold, 1986; Gibby et al., 1990). The other two failed due to a lack of flowering HORT with *P. peltatum* (HORT × PELT) or poor greenhouse conditions (such as too high humidity or temperature) for the paternal source HORT with *P. ranuncluophyllum* (HORT × RANU). Except for HORT × ZONA (Baur, 1909 and many others since), HORT × ACET and HORT × QUIN (Hondo et al., 2014, 2015), these crosses are novel and were never reported in literature before. Remarkably, in 10 cases the F_1_ offspring displayed segregation for leaf color phenotype (e.g., chlorosis). When segregation did occur, it ranged from varying levels of chlorosis to nearly green for some crosses to spanning the full range of possible phenotypes from lethal white plantlets to nearly fully green plants (Figure 4).

#### Establishment of the F_2_ Populations

For all F_1_ crosses we were able to obtain an F_2_ generation (Figure 3) with varying degrees of success, e.g., the green F_1_ “HORT × ZONA” cross used to produce the F_2_ yielded significantly more offspring as well as a slightly higher germination success than the variegated or chlorotic parents did (Table 5). The seed phenotypes for F2 crosses which were used in this study are shown in Figure 6.

**TABLE 5:** F_2_ material obtained using selection of plants from Table 1.

Cross	G	V	C	Lethal*	# seeds	Sum	Germination %	Marker(s) found	Ratio
F_2__hort_x_zona^ C^	8	1	51	34	125	94	0.78	5 FRIN, 1 ZONA	∼1:6:4
F_2__hort_x_zona^*G*^	139	3	138	20	332	300	0.84	1 FRIN, 5 ZONA	∼7:7:1
F_2__hort_x_zona^*V*^	52	4	79	48	230	183	0.76	2 FRIN, 1 ZONA, 2 FRIN, and ZONA	∼1:2:1
F_2__hort_x_acet^ C^	33	2	49	28	116	112	0.95	ACET	1
F_2__hort_x_frut^*G*^	24	3	63	0	90	90	1.00	FRIN	1
F_2__hort_x_inqu^*G*^	144	0	0	0	144	144	1.00	FRIN	1

*Chlorosis phenotypes of F_1_ parents and plant counts are given. “**G”** denotes a green plant, “**C**” a chlorotic one and “**V**” a variegated plant. For the description of the chlorosis categories see the text. **^∗^**Lethal plants are counted without the seeds that failed to germinate.*

### Primer Design and PCR Verification

We designed 11 primer-pairs targeting single accessions (e.g., genotype-specific primers) or a group of accessions (Table 6). All primer-pairs performed as expected, except BART, which amplified ARTI but not BARK. We therefore used this primer only for detecting ARTI. For gel photo documentation accompanying the primer pair evaluations we refer to Supplementary Figures 2, 3. All primers worked across a range of template DNA concentrations (0.1 ng/μl up to > 5 ng/μl). A 1/10th dilution of the extracts generally increased PCR performance.

**TABLE 6:** Primer pair details.

Primer pair name	Target(s)	Sequence 5′–>3′	Plastome region
FRIN	*P. inquinans, frutetorum and × hortorum*	AAAGGCCAGATTGGGCGGC	F: IGS and R; 5′ of rna polymerase beta subunit 2 exon
		GACGAATTCGGTCCGATTCAACAC	
ZONA	*P. zonale*	GAATTGTAATGCGGAGCTGC	F and R: IGS
		AAGAAAGAGGATATAGCCGGAC	
ACET	*P. acetosum*	GAATCCCCACCTACACTACAC	F and R: MATK exon, 3′end
		CCTTGACTAAAGCGCAATTTTG	
ACRA	*P. acraeum*	GACCCTATCTCTCTGTATTC	F and R: IGS
		TTTGGTCTCCGAAAAGAAAAGG	
ALRA	*P. alchemilloides* and *ranunculophyllum*	GGATCTTATCTATTCTCTATTC	F and R: IGS just downstream of trnK-UUU small exon
		CGATCTAGATCTAATTGTAC	
MUQU	*P. multibracteatum* and *quinquelobatum*	GGTTTCGCGTCAATTGC	F and R in flanking IGS’s of atpH, atpH exon is entirely covered by fragment
		CTGAATTTAGCTATGATTTCG	
ARID	*P. aridum*	CTGAACTGAACTCAAATGGA	F and R: in IGS, fragment contains trnH-IS and trnI-LE
		ATTGCGAGGATCCTACTTTG	
BARK	*p. barklyi*	GAAAGATCTATTCGAGTCGAG	F: in IGS, R: in intron between tnrL-UAA exons
		GGGGCCTCATTACATTAATC	
PELT	*P. peltatum*	CTCAAAAGAAGGGTAGAAGGG	F and R: in IGS’s surrounding trnS-GGA
		CCCTGTCTGCTCTTTCCAA	
TONG	*P. tongaense*	GATCTCAAAGCAAAGAGAGC	F: IGS, R: in ndhJ exon
		CTTGGCTAGTGTATACCATTTG	
BART	*P. articulatum* and *P. barklyi*	GAATCCAAAAGAAATGAAATG	F and R: IGS between atpB and rbcL
		AAAAGGAATAGGTTTTGTAG	

*Plastome regions were identified using genbank ID: DQ897681.1 (P. × hortorum, Chumley et al., 2006). F refers to forward, R refers to reverse.*

### Phenotyping and Genotyping the F_1_ and F_2_ Population for HORT × ZONA and HORT × ACET

For a full overview of the tests for all scenarios under all eight genetic models (Tables 4A,B) we refer to Supplementary Figure 5. We discuss here those crosses that demonstrated Mendelian patterns of segregation as well as the models under which this applies. We found that the F_1_ plants segregate for chlorosis, with no obvious Mendelian patterns of segregation (Table 4A and Supplementary Figure 5), but that they are otherwise phenotypically homozygous, i.e., non-segregating. When genotyping the F_1_ plants, we found that green individuals contained the *P. zonale* type plastid (ZONA), whereas chlorotic individuals contained that of *P. frutetorum/P. inquinans* (FRIN) (Tables 2, 3 plastids of “maternal origin”). A small minority (< 5%) of the plants displayed (partial) variegation and this percentage reduced, for most, as the plant aged with most settling into a single phenotype. From these we detected either the FRIN or the ZONA plastids, but as we recovered both from the F_2_ offspring (see below) they must actually have contained both. We have evaluated plastid types in all phenotypes of F_2_ offspring (structured per F_1_ cross, Figure 5) for the HORT × ZONA cross series. We found FRIN and ZONA plastid types in the F_2_ (Table 3) and, in general, F_2_ offspring always contained the same plastid as was detected in the F_1_ plant (for example, see Figure 5), except for the variegated plants. In the F_1_ HORT × ZONA variegated plants we found only one of the plastids, either FRIN or ZONA, but in the F_2_ we detected both, even once in one variegated individual (Figure 5A). We analyzed the bleached and green tissue from this plant and found that white tissue predominantly contained the FRIN type and green contained the ZONA type (Figure 5A).

When pooling the green and light green plants and treating these as one (scenarios 4 and 5) phenotype, subsequent testing for Mendelian patterns of segregation did not yield a clear pattern (Supplementary Figure 5), as was the case for three phenotypic categories. When we categorized the phenotype ratios as “affected” or “not affected,” we saw that they matched those expected under either a one- or two-gene model for all crosses assuming lethal interactions are also possible between alleles. The populations where the ratios conformed to the one gene model are F_2_ HORT × ZONA^*V*^ and F_2_ HORT × ZONA^*C*^. The segregation ratios in this “affected vs. not-affected” analysis pointed to one lethal combination of alleles and two combinations that yield viable or affected plants. When pooling light green and yellow plants and subsequently testing for Mendelian patterns of segregation, a pattern emerges for the F_2_ HORT × ZONA^*V*^ and the F_2_ HORT × ZONA^*G*^ populations (scenario 2). In contrast, when analyzing the observations for the F_2_ HORT × ZONA^*C*^ plants there did not appear to be a pattern. The patterns for the F_2_ HORT × ZONA^*V*^ and the F_2_ HORT × ZONA^*G*^ populations did point to a genetic difference in the F_1_ population (and therefore also in the F_0_ populations). With the green populations following the one gene model whereby the F_1_ was Aa × AA.

The ratios for the plants phenotyped for F_2_ seeds and their corresponding possible underlying genetic models are listed in Table 4A. We deduced that there were likely one (in HORT × FRUT and in HORT × ZONA) and two loci (in HORT × ACET) interacting in this phase of plant development. Given that the phenotypic ratios under scenarios 1–4 did are similar to, but not exactly what would expected when of one, two, or three genes interact. We suspect that more complex interactions, possibly involving more than two or even three genes, played are role or that the loci involved are linked in some cases with aggravating or moderating effects of linked loci. This appears especially to be the case for HORT × ACRA where ratios under scenarios 1–4 are: ∼2:1:2:20 (3 loci); ∼2:1:2 (2 loci); ∼1:1:10 (3 loci), and ∼1:12 (3 loci), respectively (see Supplementary Figure 5 for more details).

#### Positive Controls

Our positive controls HORT × INQU and HORT × FRUT yielded 100% green plants in the F2. In the F_2_ this was maintained for HORT × INQU for both plant and seed phenotypes, but surprisingly, the F_2_ of HORT × FRUT displayed segregation for chlorosis and seed phenotypes (Figures 4A and 6) indicative of the one gene model of segregation with a heterozygous parent with possible lethal combinations expressed in the pre-seedling phase as well (Table 4A and Figure 6).

#### Genotyping Phylogenetically More Distant F_1_ Hybrids

We recovered two plastid types in the offspring of F_1_ of HORT × QUIN (Table 2). We found segregation for chlorosis and detected both the FRIN type as well as the MUQU type plastids in the offspring. None of these plants were fully green. In the F_1_ HORT with *P. aridum* (HORT × ARID) we found segregation for chlorosis, with the majority of offspring lethal and one plant surviving a full season. For F_1_ HORT × ARID We detected FRIN and ARID plastids in the offspring. In the F_1_ HORT × ALCH, F_1_ HORT × TONG, F_1_ HORT × ACRA, F_1_ HORT × MULT and HORT × BARK, we detected only the paternal plastids (Supplementary Figure 2). This is similar to the F_1_ HORT × ACET cross in that we detected only one type in the offspring suggesting lethal interactions with the FRIN type plastid. In the F_1_ HORT × ARTI cross we find segregation for chlorosis and no correlation between phenotype and genotype, we detected both the FRIN and ARTI type plastids. For an overview of all the results (see Table 2).

## Discussion

We show that biparental inheritance occurs throughout the section and that hybridization is relatively easy, both observations have important implications for interpreting current concepts of *Pelargonium* section *Ciconium* evolution. This study further demonstrates that using multiple interspecific crosses can be used to gain insight into the genetics underlying organelle management and expression, potentially uncovering drivers of speciation. Our studies expand on the two-interacting gene model found to regulate plastid inheritance in *Ciconium* which was inferred 50 years ago by Tilney-Bassett et al. (1989b, 1992). While a limited number of crosses between *P. × hortorum* and section *Ciconium* have been previously reported (e.g., Hondo et al., 2014, 2015), we have greatly expanded on this by covering nearly all of the CWR in the section including those that are phylogenetically more distantly related.

### Biparental Inheritance of Plastids and Evolutionary Implications

We have found maternal (*P. frutetorum/inquinans*; FRIN) and paternal (other *Ciconium* plastid types) inheritance in nearly all our offspring indicating that the ability to inherit and express more than one plastid is the rule rather than the exception in *Pelargonium* section *Ciconium*. Even though it was demonstrated before on a limited scale (Baur, 1909; Tilney-Bassett et al., 1992; Weihe et al., 2009), it was never demonstrated to be so ubiquitous. This has important implications for the study of *Ciconium* speciation as bi-parental inheritance may provide an escape from the acquisition of deleterious plastid mutations (Mullers ratchet), because there is the possibility for an additional plastome types to occur in the individual plant. Also, it may allow to occupy new niches quicker and perhaps even allow populations that have become separate in space and time to reconnect (Greiner et al., 2011; Apitz et al., 2013; Greiner and Bock, 2013; Greiner et al., 2015; Barnard-Kubow et al., 2016, 2017; Sobanski et al., 2019).

### Plastid Effects

We have found evidence that in our crosses the FRIN plastid caused bleaching in the HORT × ZONA crosses and that it was possibly lethal for the HORT × ACET cross given the absence of any offspring containing FRIN. The observation that ZONA plastids caused less chlorosis than FRIN in these types of crosses is not new in itself and this study confirms what was already hinted at by Tilney-Bassett and Almouslem (1989a) and more recently confirmed by Weihe et al., 2009 who observed that the “inquinans plastid” caused bleaching. The F_1_ HORT × ZONA plants were, in some cases viable when containing the FRIN plastid allowing us to evaluate the effects of both plastid types in subsequent generations. As to which part of the plastome is the root cause we can only speculate, but a number of genes have been demonstrated to be under selection in the Geraniaceae plastomes (Shikanai et al., 2001; Blazier et al., 2016a, b; Ruhlman et al., 2017; Weng et al., 2017; Ruhlman and Jansen, 2018). More surprising was the find that the F_2_ HORT × FRUT showed a segregation for chlorosis, even though the F_1_ did not. This hints at a slight incompatibility between the FRIN type plastid and either the HORT or FRUT parent. This is surprising given that we cannot distinguish the plastids. Therefore, given the segregation ratios (Table 4A), one nuclear gene, either originating from HORT or FRUT, must be slightly divergent and must be responsible for this effect. Given that this segregation was not the case for The HORT × INQU F_2_ population and no segregation occurs when selfing HORT, we deduce that one of the alleles originating from FRUT was responsible.

### F_2_ Segregation Pointing to Two or Three Epistatically Interacting Genes

We demonstrate, in a second generation series of plants that, irrespective of plastid type, there was segregation for chlorosis. Chlorotic phenotypes of the F_2_ did not appear to show Mendelian inheritance patterns under a one or two allele model in all cases. However, nuclear alleles must be involved because the plastid backgrounds are the same for each plant (Stubbe, 1958, 1959, 1989; Amoatey and Tilney-Basset, 1994; Barr and Fishman, 2010; Li et al., 2013). For the F_2_ HORT × ZONA^*V*^ population both the one gene model and the two gene model did seem to be equally good at explaining the results. The observed numbers conformed well to the F_1_ HORT × ZONA^*V*^ population being heterozygous. As outlined above, ratios for the three phenotypic categories do not shed much light on the underlying genetics, but when we categorize the phenotype ratios in a binary way, “affected or not affected,” we see the ratios for all crosses matching or approaching ratios for phenotypes that resemble the situation where one combination is lethal and two combinations of alleles are not. For the HORT × ZONA^*C*^ population the ratio is more akin (10:1 under the two phenotypes scenario 5, Supplementary Figure 5A) to the ratio’s expected (9:1 under the two phenotypes scenario 4, Supplementary Figure 5A) under a two gene interaction model whereby heterozygous combinations are lethal and the homozygous combinations of at least one allele are not. The ratios for the HORT × ACET cross hint at a possible trihybrid segregation, whereby two alleles interact in a lethal way, because of the following reasoning: If segregation was perfect we would expect the following phenotypic ratio’s under the three gene model; 27:9:9:9:3:3:3:1 but we observe 25:9:1:5 under the four phenotypes scenario 1 (Supplementary Figure 5A). For this pattern to occur we would have to assume there are two alleles that interact in a lethal way, causing the deviation from the expected ratio’s, but also that there is a third allele which in turn moderates some of these effects or may cause extra lethality.

The ratios of CNI phenotypes observed in the seeds points to a similar type of interactions further explaining why we observe sometimes skewed segregation ratios. In the case of the HORT × ACET cross we observe mendelian segregation of under gene models b, f, h (25:75 phenotype ratios under the one, two, and three gene models) with the majority of the individuals being lethal. When we view the ratios of all phenotypes for HORT × ACET and HORT × ACRA (10:3:2:1 and 20:2:1:2, respectively, Supplementary Figure 5B) these, similarly as for the seedlings evaluated, reminiscent of ratios for the two gene model whereby heterozygous combinations are lethal and the homozygous combinations of at least one allele are not. Thus, combining the observations of both seed and seedling phase of plant development, would yield for the HORT × ACET cross a series of at least five loci involved in development and expression of organelles. For the HORT × FRUT at least two loci would be required to explain the observations, one acting in each stage of development we studied.

### Model of > 3 Interacting Nuclear Genes

The observation that the HORT × ACET cross needs two and a three gene model to explain the observed patterns may indicate that those crosses which consist of combination that are phylogenetically further removed from HORT may be subject to the effects of more than three genes. As mentioned above CNI plays a role in embryo and fruit development as well. This in turn could point to a more complex model of genetic interactions involving more loci than we thus far proposed. The machinery for synthesis and management of organelles consists of numerous PPR genes that each act during a different step of these processes (Barkan and Small, 2014; Börner et al., 2015; Zhang and Lu, 2019). These can perhaps be viewed as a genetic “block chain” whereby no mismatch of combinations is allowed in order to result in a viable, green and self-sustaining plant. In our interspecific crosses there were ample opportunities for these mismatches to occur. While we have no hard evidence for this we do see from the numbers of plants recovered from our crossing attempts decreases with increased phylogenetic distance. In other words for the plants from the crosses of e.g., HORT × ARID we obtained one plant only using the same effort as was invested in the other crosses. This one plant may represent the rare, fortunate gene combination that allows the individual to survive under ideal conditions, while all other combinations are lethal. Given that phylogenetically close crosses (HORT × FRUT, HORT × ZONA, HORT × ACET) require the one, two, or three gene model with the assumption of lethality to explain the phenotypic ratios for both the seedling and seed phase we evaluated, we may just be viewing the tip of the iceberg for the phylogenetically more distant crosses. Generally, genes thought to be involved in chloroplast management and expression are Whirly genes (Maréchal et al., 2009; Isemer et al., 2012; Krupinska et al., 2014, 2019), involved in importing proteins into chloroplasts (Krausea et al., 2005; Chateigner-Boutin et al., 2008; Mackenzie and Kundariya, 2019), and PPR genes, acting at the level of RNA editing (Takenaka et al., 2013; Wang et al., 2016a, b; Rojas et al., 2019; Small et al., 2020). These genes are good candidates to study in *Pelargonium* and a closer study of the proteins they encode for as well as the type of RNA editing taking place, may explain both biparental inheritance as well as early stage processes of speciation.

### Data Quality

Our approach to phenotyping contains a number of potential sources of error possibly obscuring more nuanced phenotypic differences. We evaluated the seedlings at two points in time to correct for differences in development phase and possible environmental effects on the stability of the phenotypes. Differences in ambient temperature at each point can, potentially severely, affect the expression of chlorosis (pers. observations all authors). Furthermore, the interpretation of the photos, while allowing for reviewing the phenotyping afterward is subject to interpretation. Defining a plant as “affected” or not is sometimes context dependent. In the initial germination phase seedlings were germinated under controlled conditions and all at the same time to insure that we were comparing plants in equal phases of development. Great care was taken to make sure the photos of each set were taken at the same day to reduce chance of observing changed phenotypes when environmental conditions change. A further reduction of errors in interpretation can, in the future, be achieved by germinating seeds under even more controlled conditions and using automated imaging software, for interpretation of chlorotic phenotypes (see for an example of this approach Flood et al., 2020).

Seed phenotypes in *Pelargonium* related to CNI have not been studied before. We have chosen very clear-cut categories and in doing so may have underestimated the actual level of CNI. Nevertheless, our phenotypes are reminiscent of what is regularly encountered in relation to mutated organelle expressing PPR genes in *Arabidopsis thaliana* (Chi et al., 2008; Du et al., 2017; Zhang et al., 2017). Finally, in some cases we found a discrepancy in plastid types detected, between parents and the offspring of the variegated plants. Probably, variegated plants are able to manage and express both plastids and subsequently one type is outcompeted but not completely removed. This competition was demonstrated in *Oenothera* and occurs at a cellular level (Sobanski et al., 2019).

### Crossings

In our study we have obtained at least one individual F_1_ hybrid plants for the majority of interspecific crosses attempted (except for *P. ranunculophyllum*). Most were obtainable from seed showing high compatibility of the genomes and plastids. We attribute the two unsuccessful crosses to suboptimal greenhouse climate conditions as we observed that for a pollination to be successful abiotic factors such as climate and humidity are important (reviewed by Lohani et al., 2020). The chance to obtain a (viable) F_1_ plant further roughly correlates with previously published plastome based phylogenetic distances (Figure 1).

Our approach in this study is reminiscent of the study recently published by Flood et al. (2020) who used cybrids to study the effects of different plastids types in equal nuclear genomic backgrounds. We have used F_1_ generation crosses which, though different from the cybrids in the sense that the nuclear genome is hybrid, is still uniform and allows us to study the effects of different types of organelles. Our approach is different that this study focuses more on an evolutionary, rather than at the population level as was the case in Flood et al. (2020).

## Conclusion and Future Applications

The insight from this study further open up possibilities for breeding of currently available *Pelargonium* cultivars with their crop wild relatives. Now we could conceivably start breeding in plastids that, for instance, perform better in warmer/colder/dryer climates allowing for the adjustment of cultivars to different climates (Deng et al., 2004; Cortés and Blair, 2018; Westerbergh et al., 2018) and other abiotic factors (Mezghani et al., 2019; Wang et al., 2019; Ribera et al., 2020; Singh et al., 2020). Especially, photosynthesis would be an interesting trait to focus on as differences between the species are, likely, more dramatic than those observed between the different populations of *A. thaliana* which has been the focus so far when studying the effects of plastid types and photosynthetic efficiency (Flood et al., 2011; Cruz et al., 2016; Flood et al., 2020). The fact that different types of plastids have a different effect in a similar nuclear background means that breeding efforts that wish to incorporate crop wild relatives to increase genetic diversity or introduce new traits should consider the organellar background of the material as well. Knowing the effects can aid in making more informed decisions as to which species to attempt a cross with and which not. This then can lead to more focused and mores successful breeding attempts.

## Data Availability Statement

The original contributions presented in the study are included in the article/Supplementary Material, further inquiries can be directed to the corresponding author.

## Author Contributions

FCB, FTB, RCS, and MES conceived the study. FCB carried out the analysis. FCB and FTB wrote the manuscript. FCB, SP, and JWK did the laboratory work PCR. MS-S did laboratory work embryo rescue. All authors read the draft and gave feedback.

## Conflict of Interest

RCS and MS-S were employed by the Syngenta Seeds BV, Netherlands. The remaining authors declare that the research was conducted in the absence of any commercial or financial relationships that could be construed as a potential conflict of interest.

## Footnotes

1 http://biotools.nubic.northwestern.edu/OligoCalc.html

## Supplementary Material

Supplementary Figure 1 Diagnostic PCR targeted on HORT × ZONA and HORT × ACET.

Supplementary Figure 2 Gel images other HORT × *Ciconium* F_1_ crosses displaying plastid types as identified using PCR.

Supplementary Figure 3 PCR mastermix and thermo profile.

Supplementary Figure 4 Overview of representative hybrid plants of F1 plants obtained in this study.

Supplementary Figure 5 Full overview of all scenarios and gene models tested.

Supplementary Figure 6 **(A–K)** Representative phenotypes for all F1 plants created in the course of this study.
