# Supplementary material for: Interspecific Hybrids Between Pelargonium × hortorum and Species From P. Section Ciconium Reveal Biparental Plastid Inheritance and Multi-Locus Cyto-Nuclear Incompatibility
Source: Front Plant Sci. 2020 Dec 18;11:614871. doi: 10.3389/fpls.2020.614871 (PMC7775418; doi:10.3389/fpls.2020.614871)
Supplement: Supplementary Figure 1 — Diagnostic PCR targeted on HORT × ZONA and HORT × ACET. [file Presentation_1.PPTX]

## Slide 1
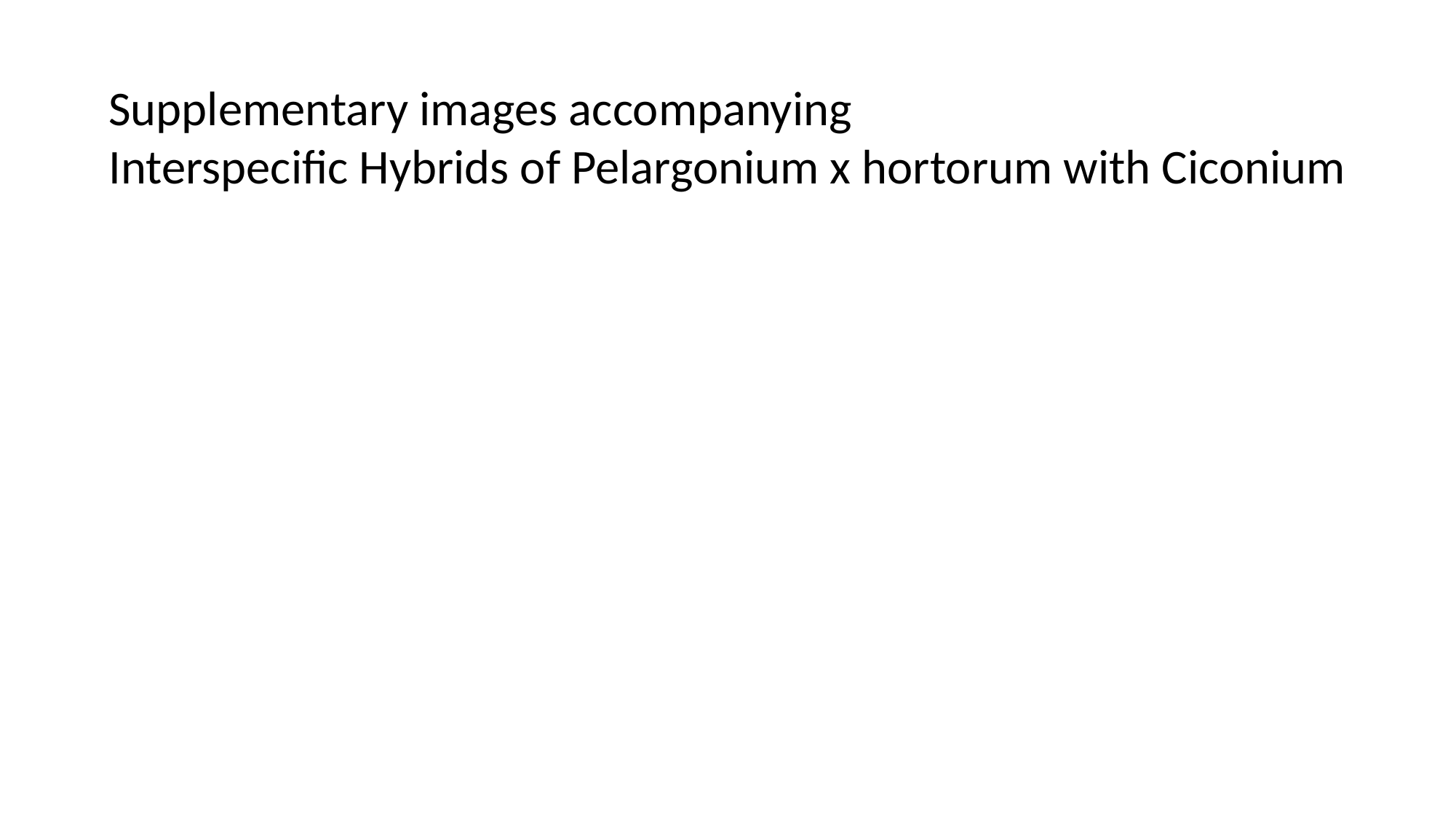

Supplementary images accompanying
Interspecific Hybrids of Pelargonium x hortorum with Ciconium

## Slide 2
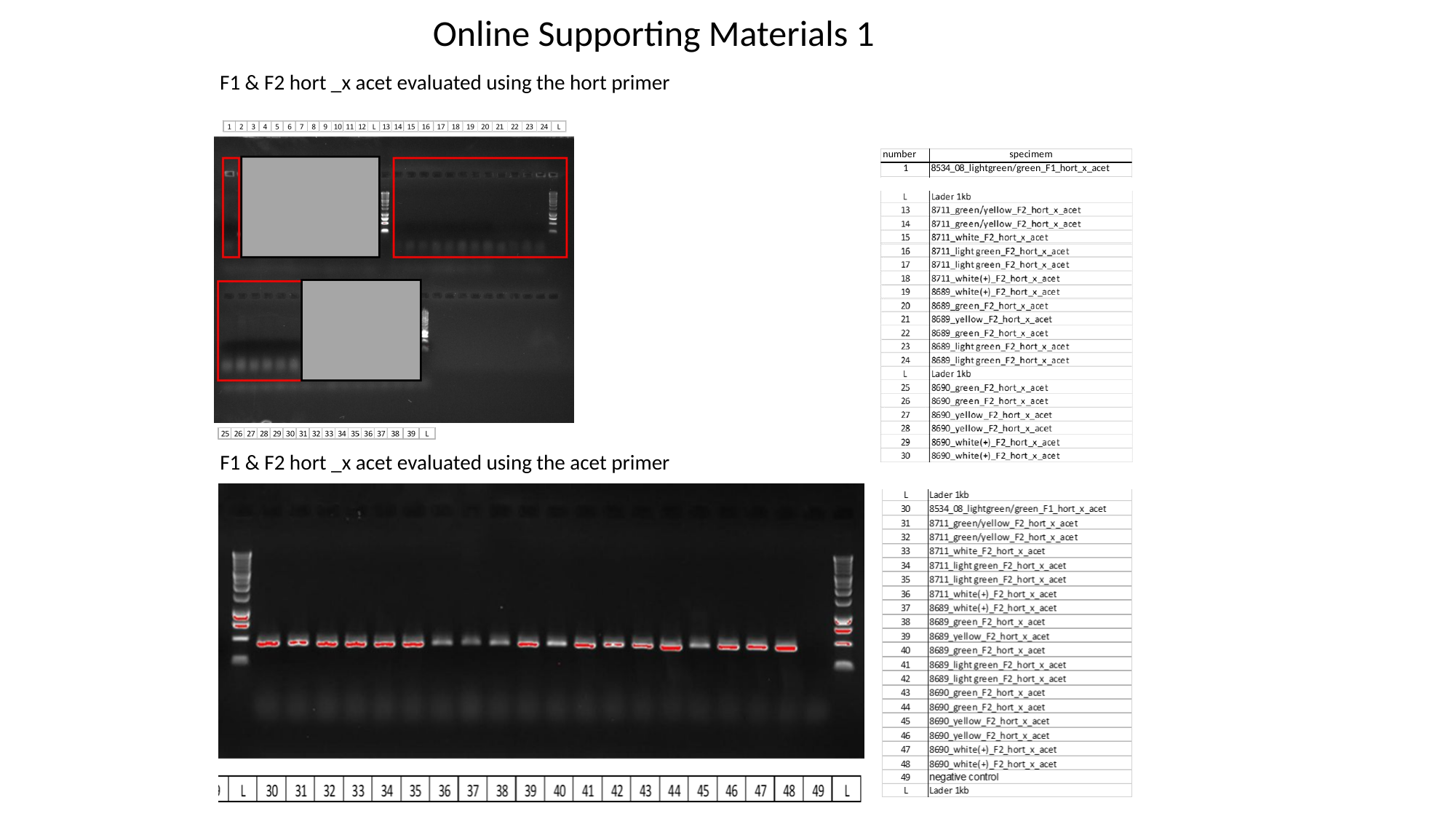

Online Supporting Materials 1
F1 & F2 hort _x acet evaluated using the hort primer
F1 & F2 hort _x acet evaluated using the acet primer

## Slide 3
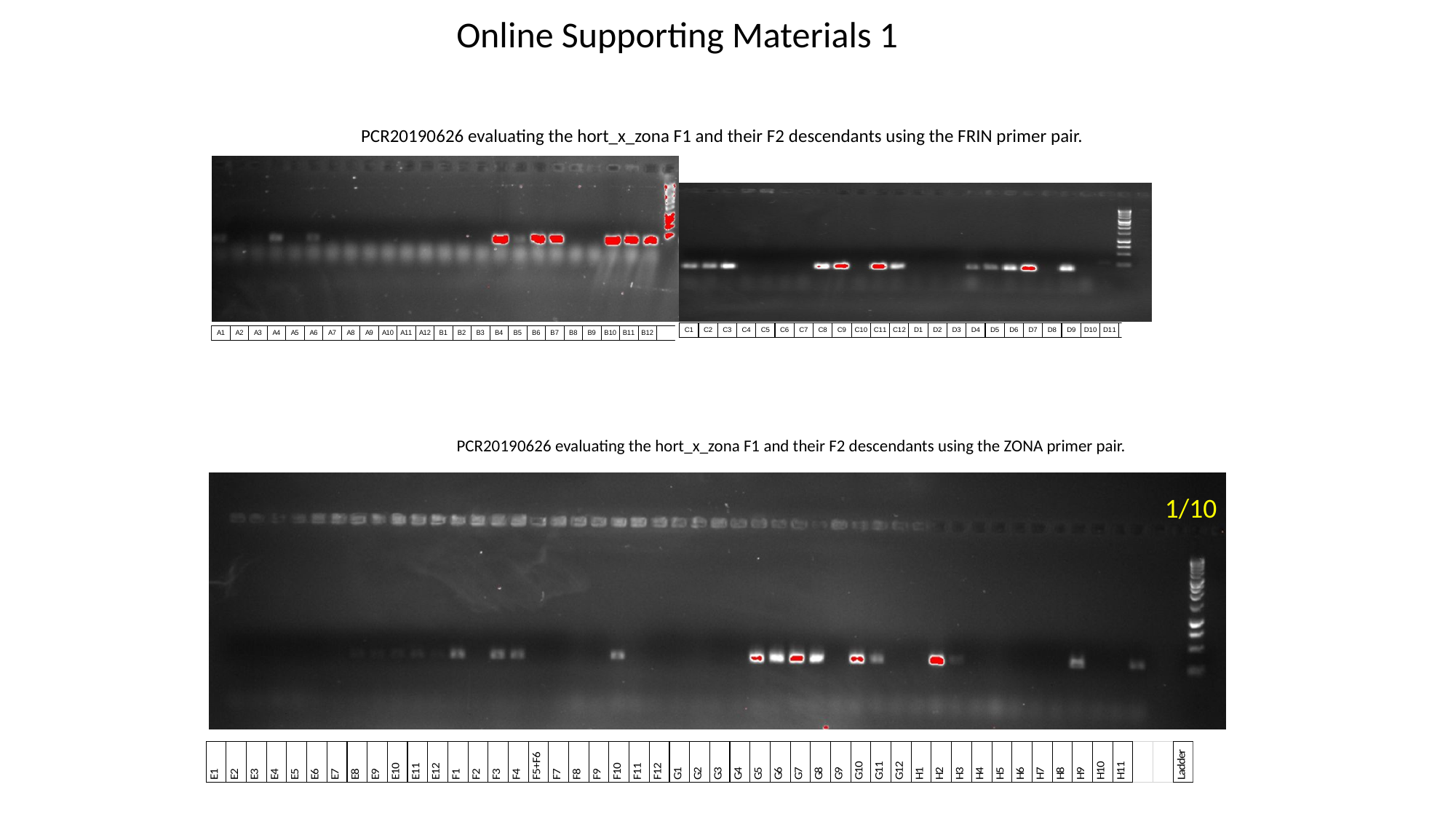

Online Supporting Materials 1
PCR20190626 evaluating the hort_x_zona F1 and their F2 descendants using the FRIN primer pair.
PCR20190626 evaluating the hort_x_zona F1 and their F2 descendants using the ZONA primer pair.
1/10

## Slide 4
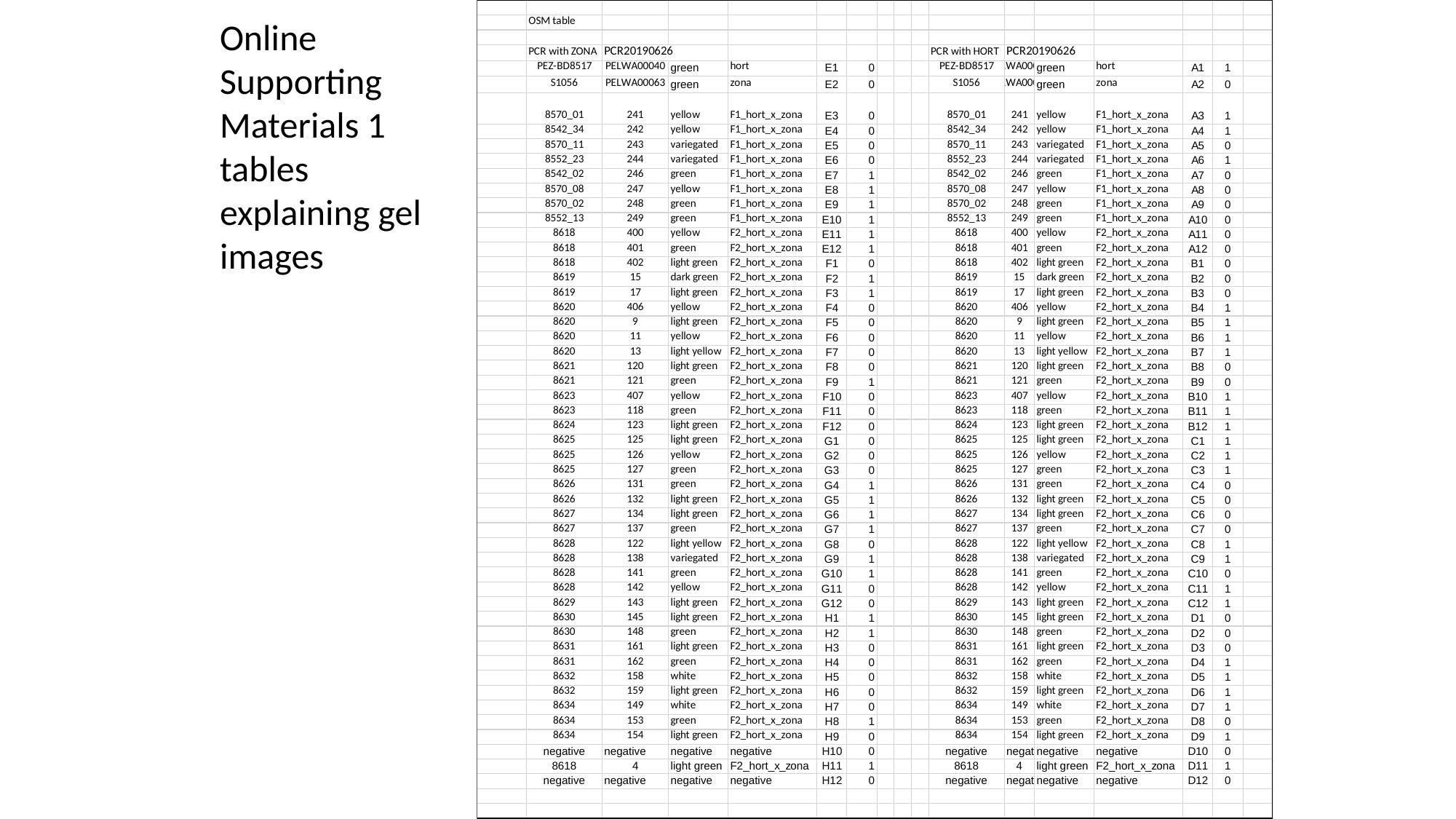

Online Supporting Materials 1 tables explaining gel images

## Slide 5
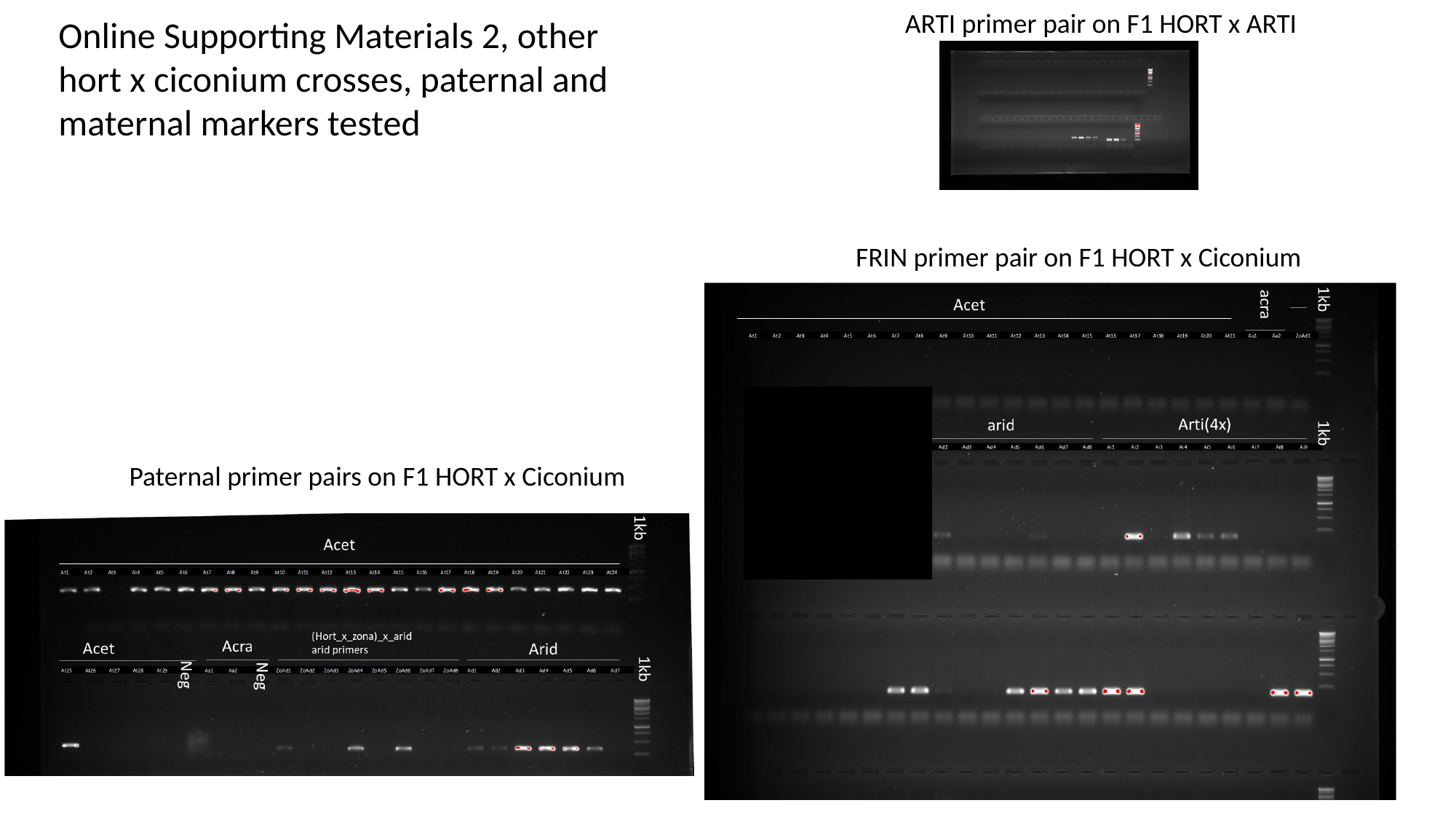

ARTI primer pair on F1 HORT x ARTI
Online Supporting Materials 2, other hort x ciconium crosses, paternal and maternal markers tested
FRIN primer pair on F1 HORT x Ciconium
Paternal primer pairs on F1 HORT x Ciconium

## Slide 6
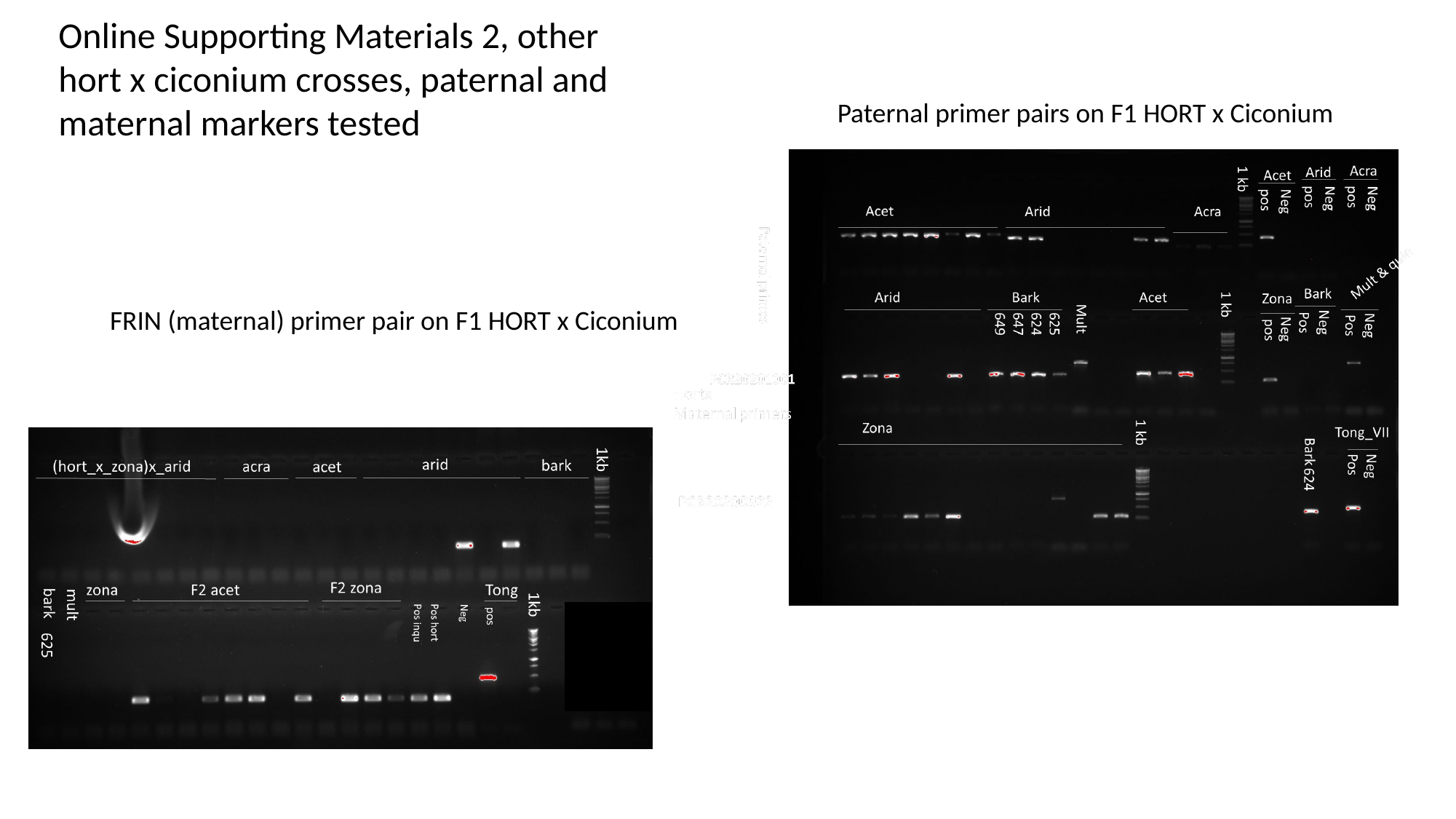

Online Supporting Materials 2, other hort x ciconium crosses, paternal and maternal markers tested
Paternal primer pairs on F1 HORT x Ciconium
FRIN (maternal) primer pair on F1 HORT x Ciconium

## Slide 7
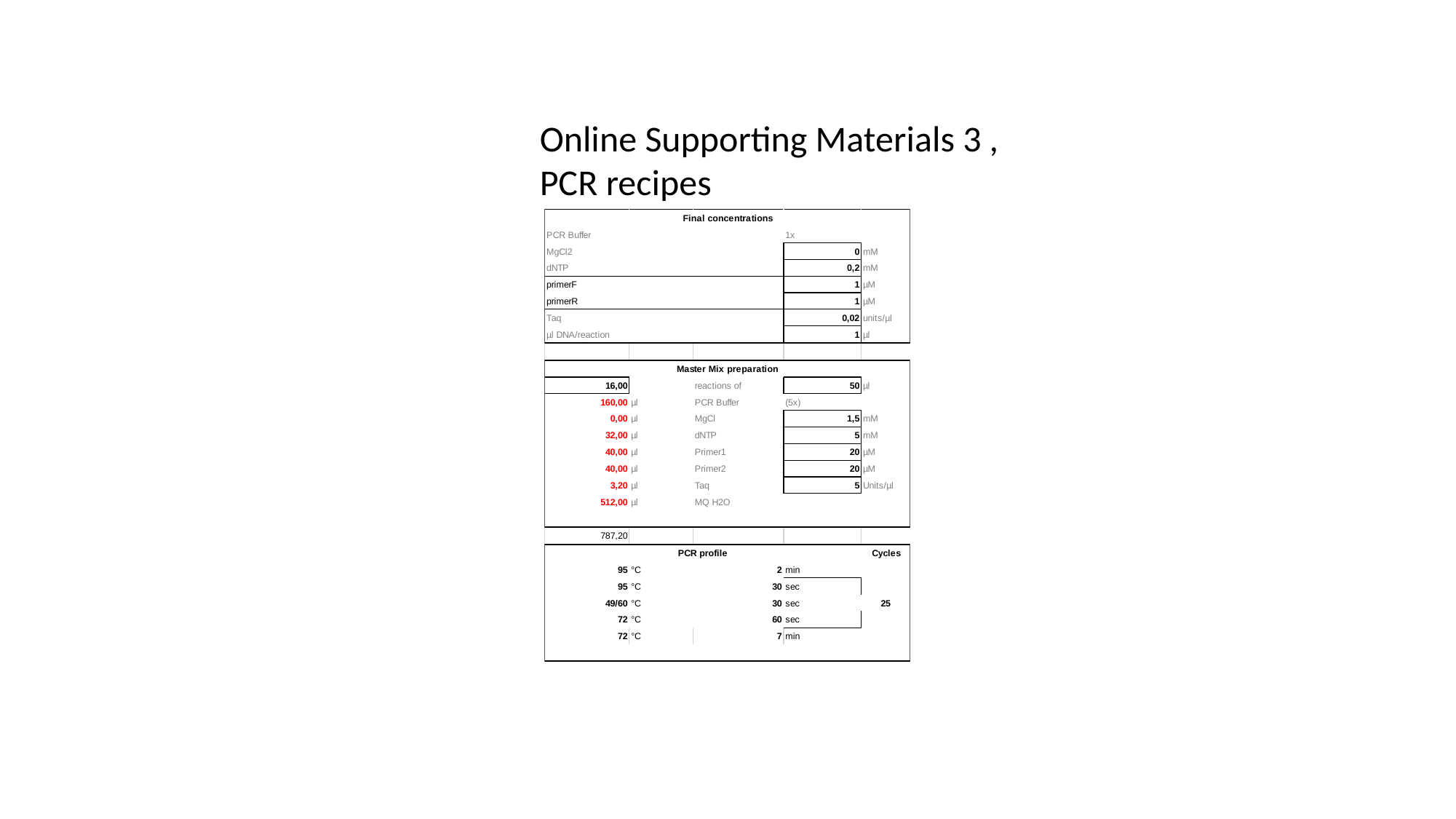

Online Supporting Materials 3 , PCR recipes

## Slide 8
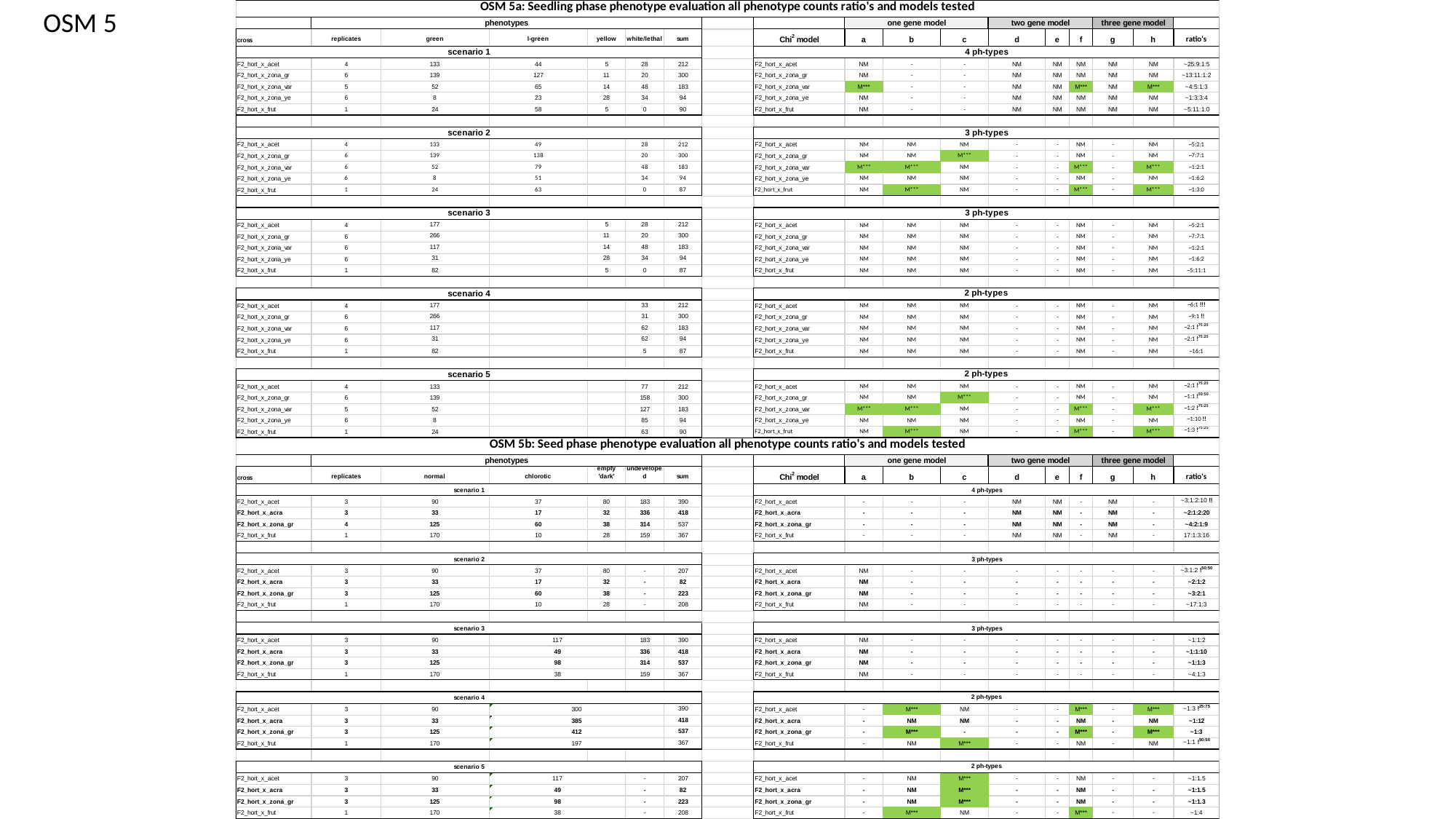

OSM 5

## Slide 9
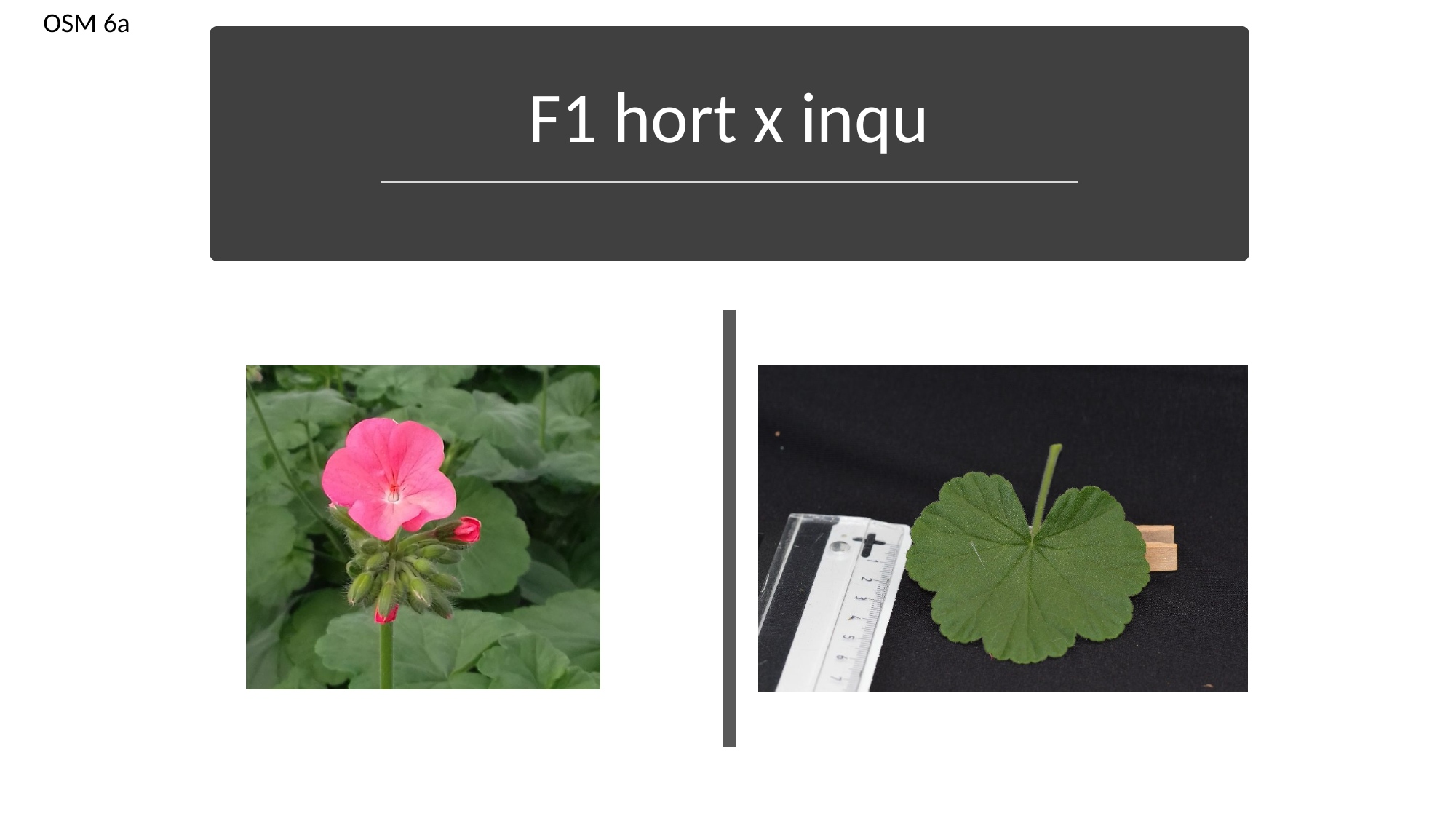

OSM 6a
# F1 hort x inqu

## Slide 10
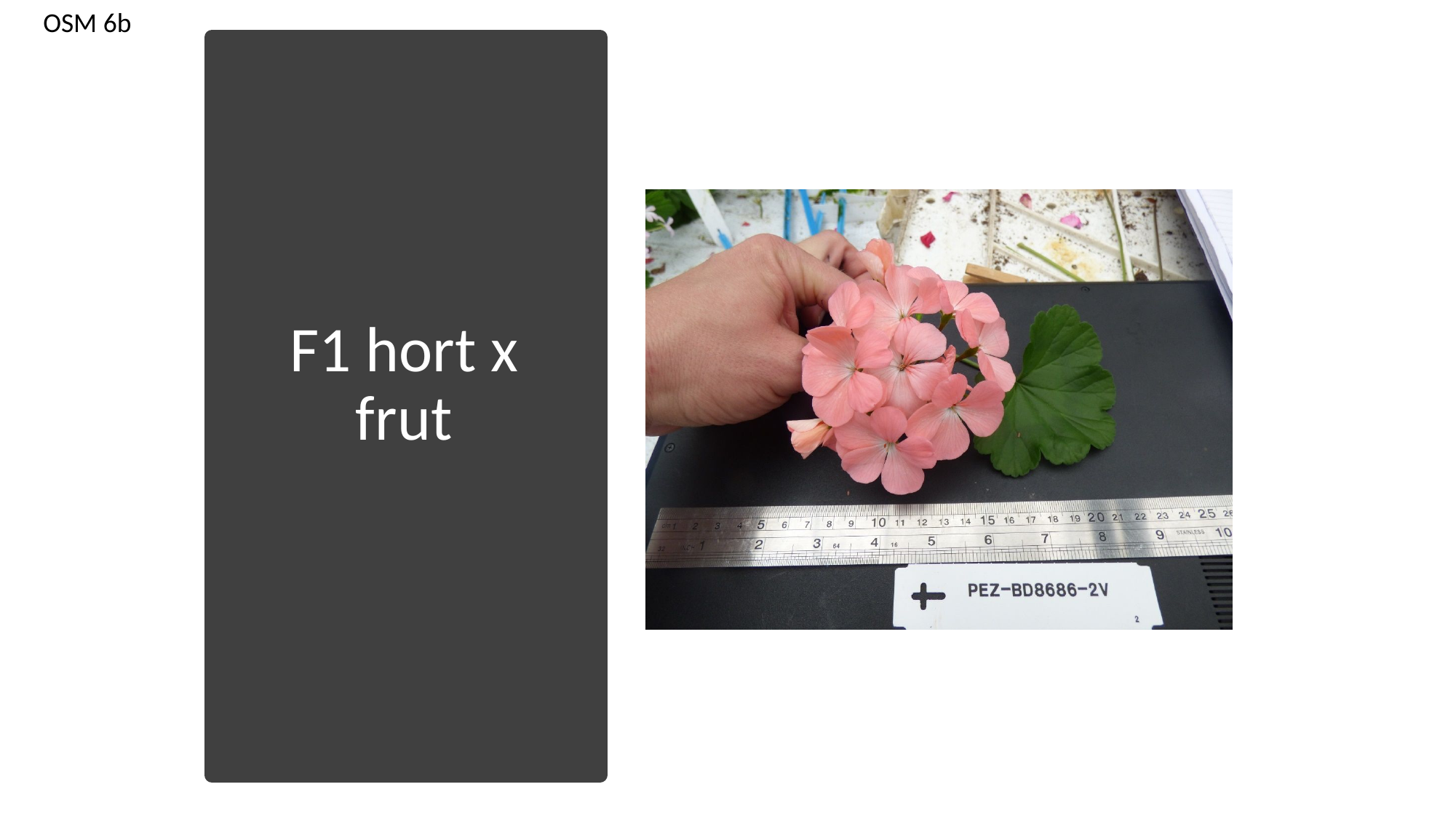

OSM 6b
# F1 hort x frut

## Slide 11
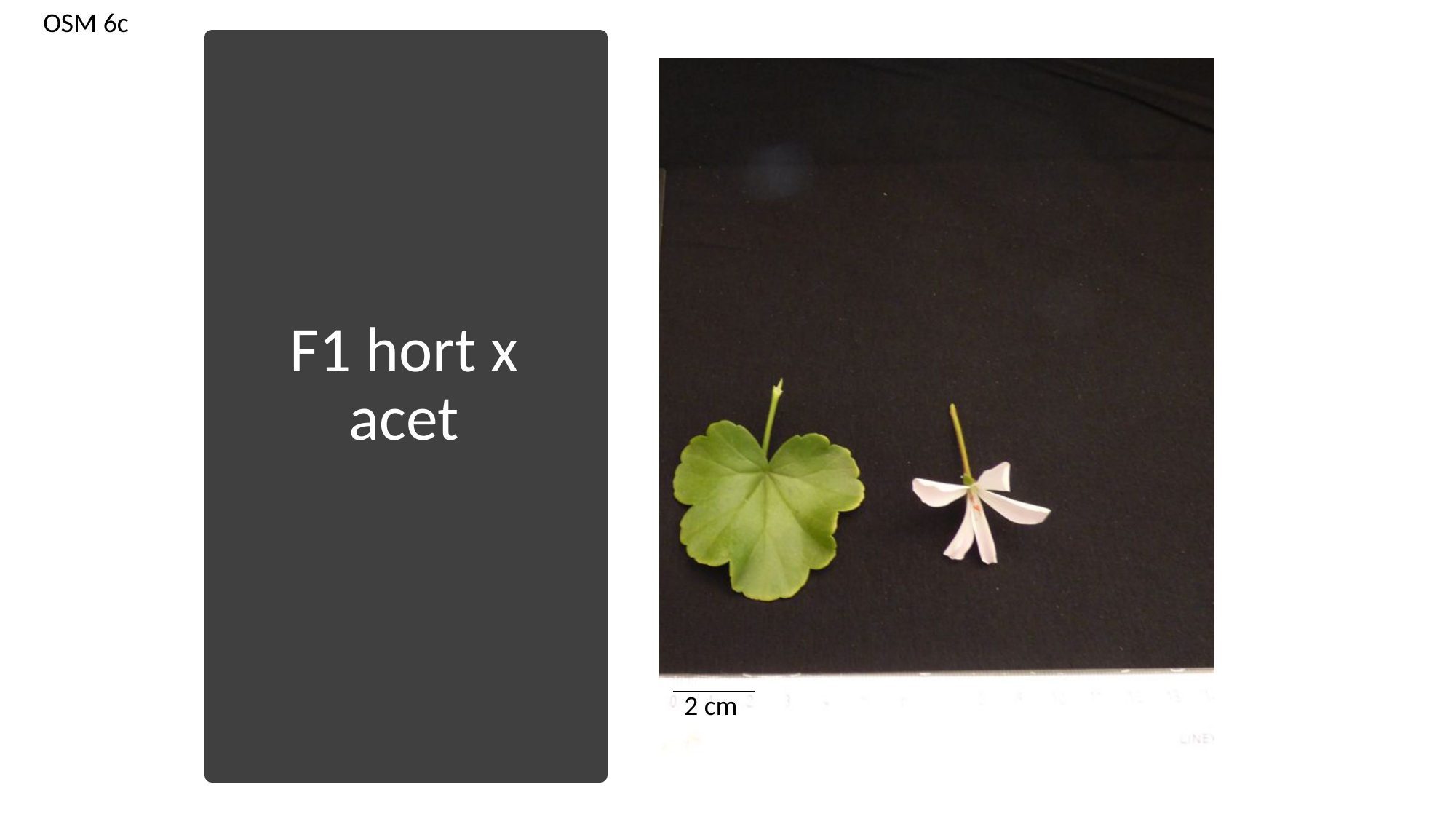

OSM 6c
# F1 hort x acet
2 cm

## Slide 12
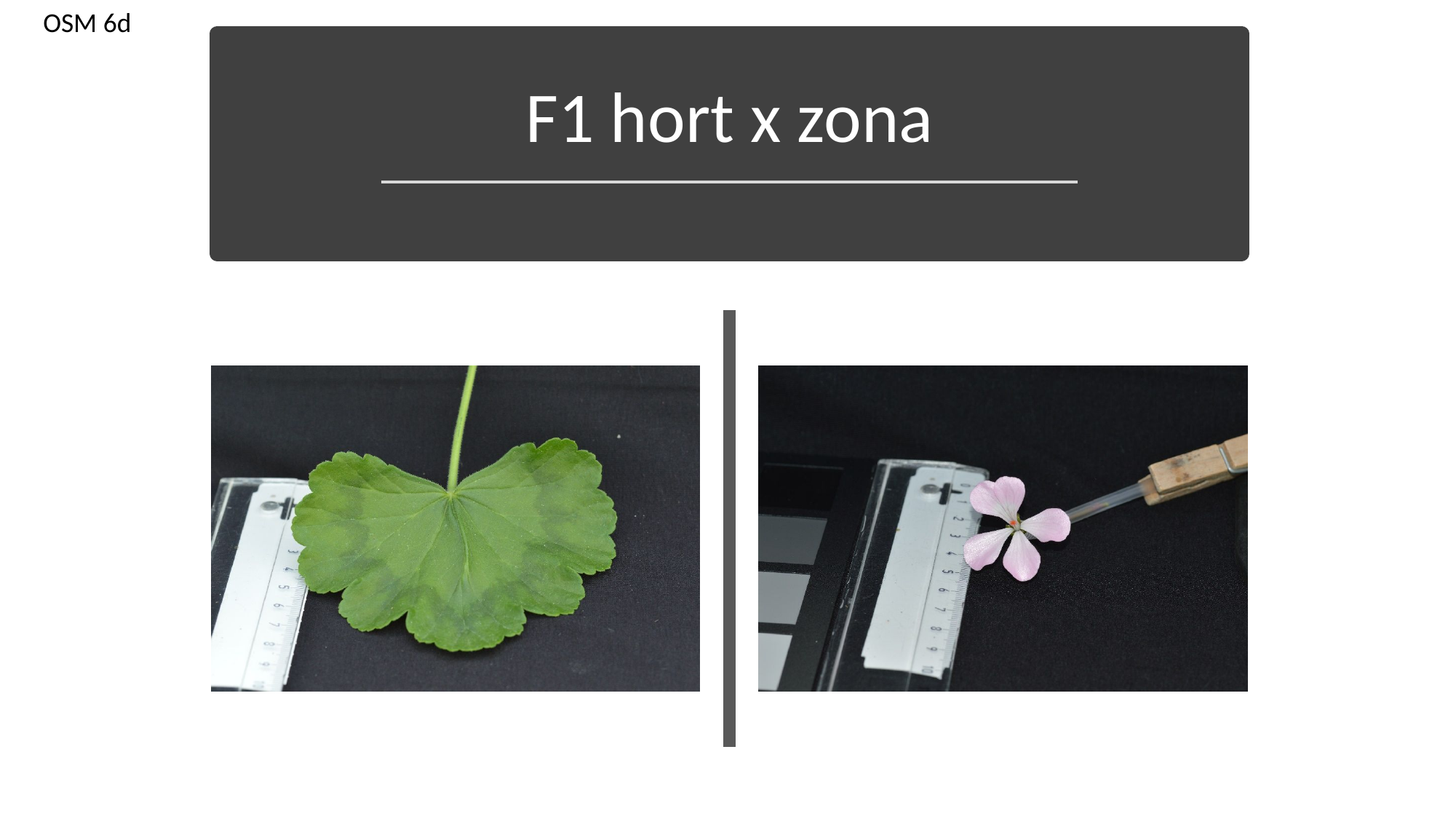

OSM 6d
# F1 hort x zona

## Slide 13
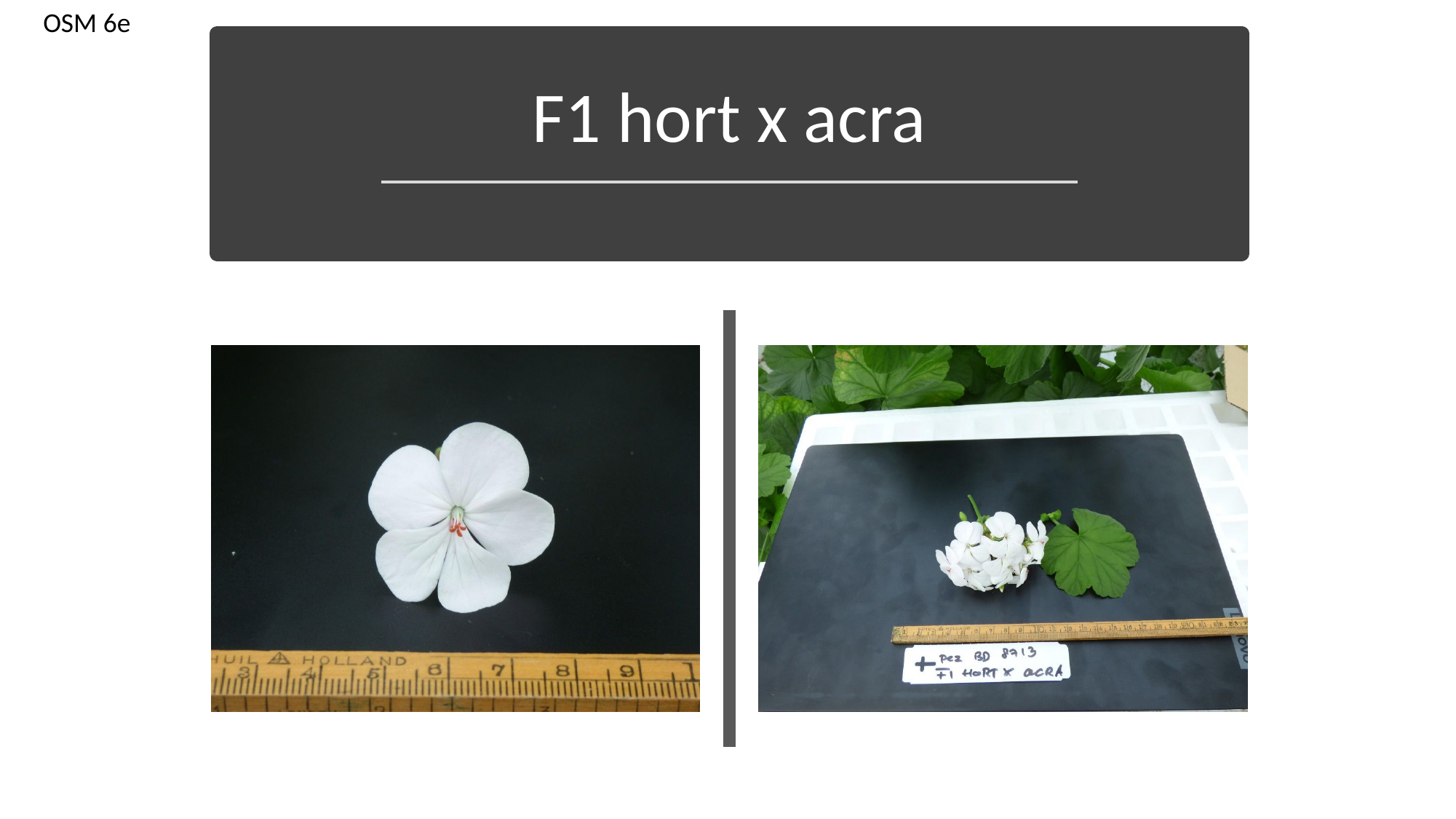

OSM 6e
# F1 hort x acra

## Slide 14
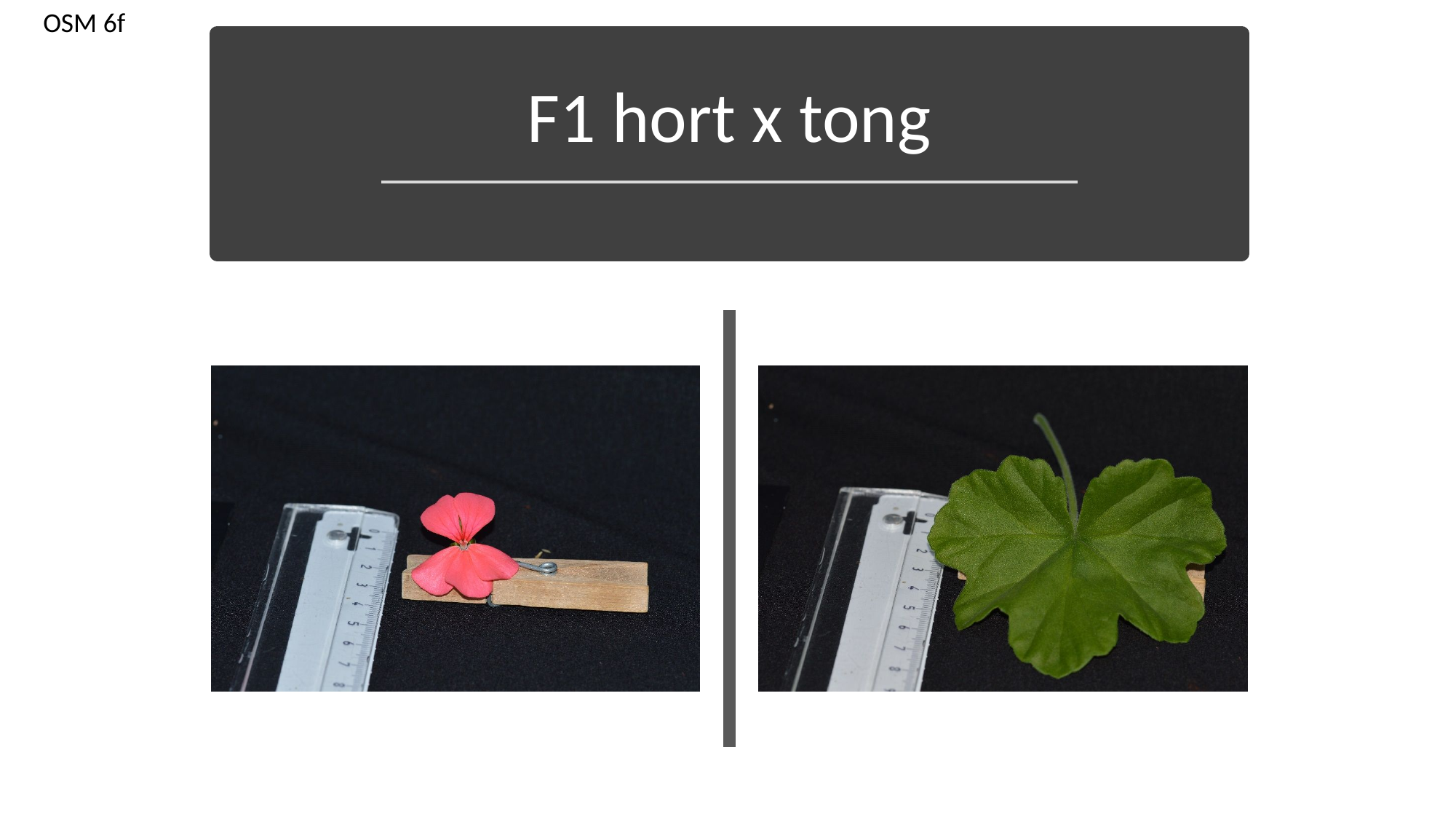

OSM 6f
# F1 hort x tong

## Slide 15
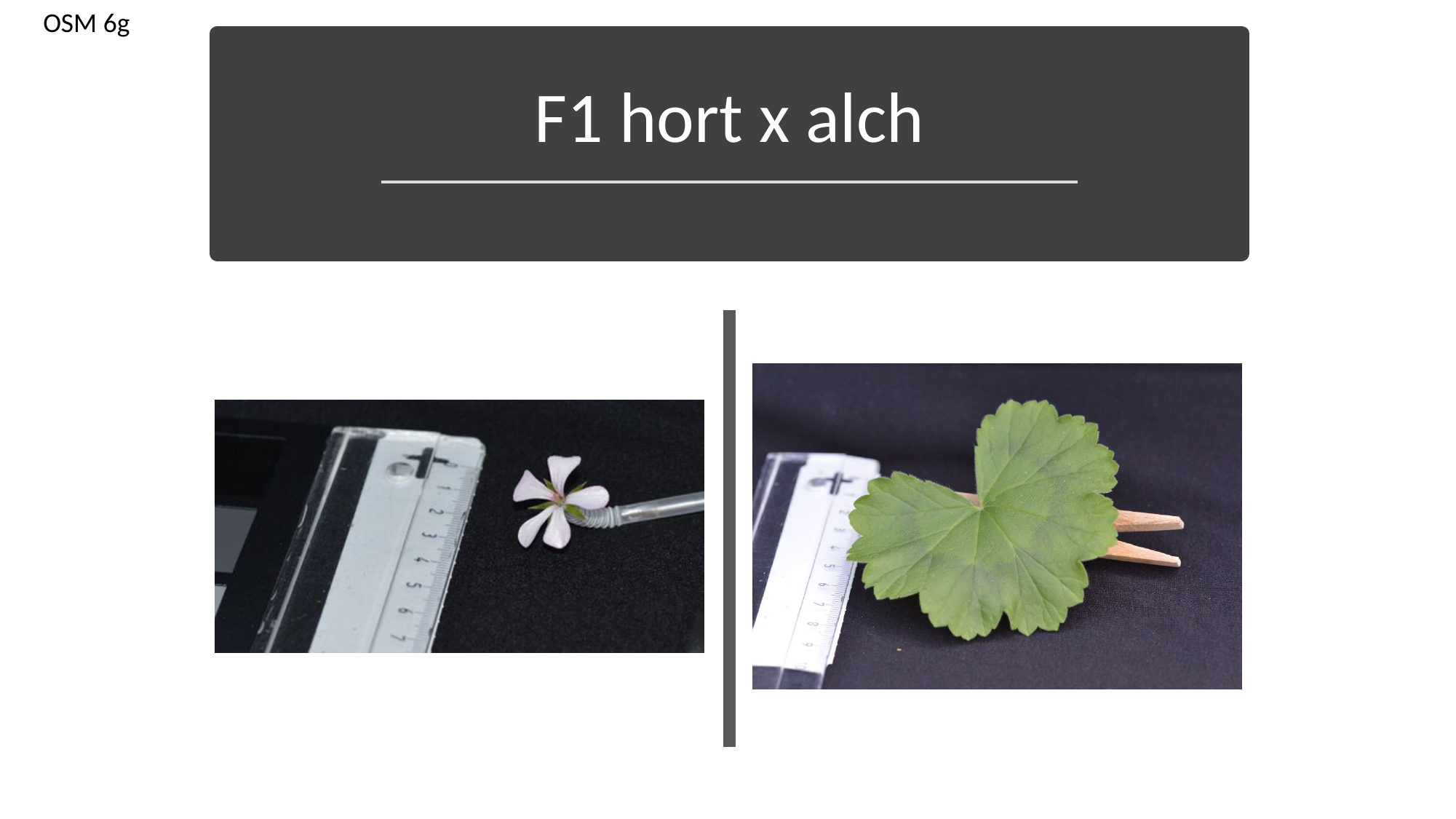

OSM 6g
# F1 hort x alch

## Slide 16
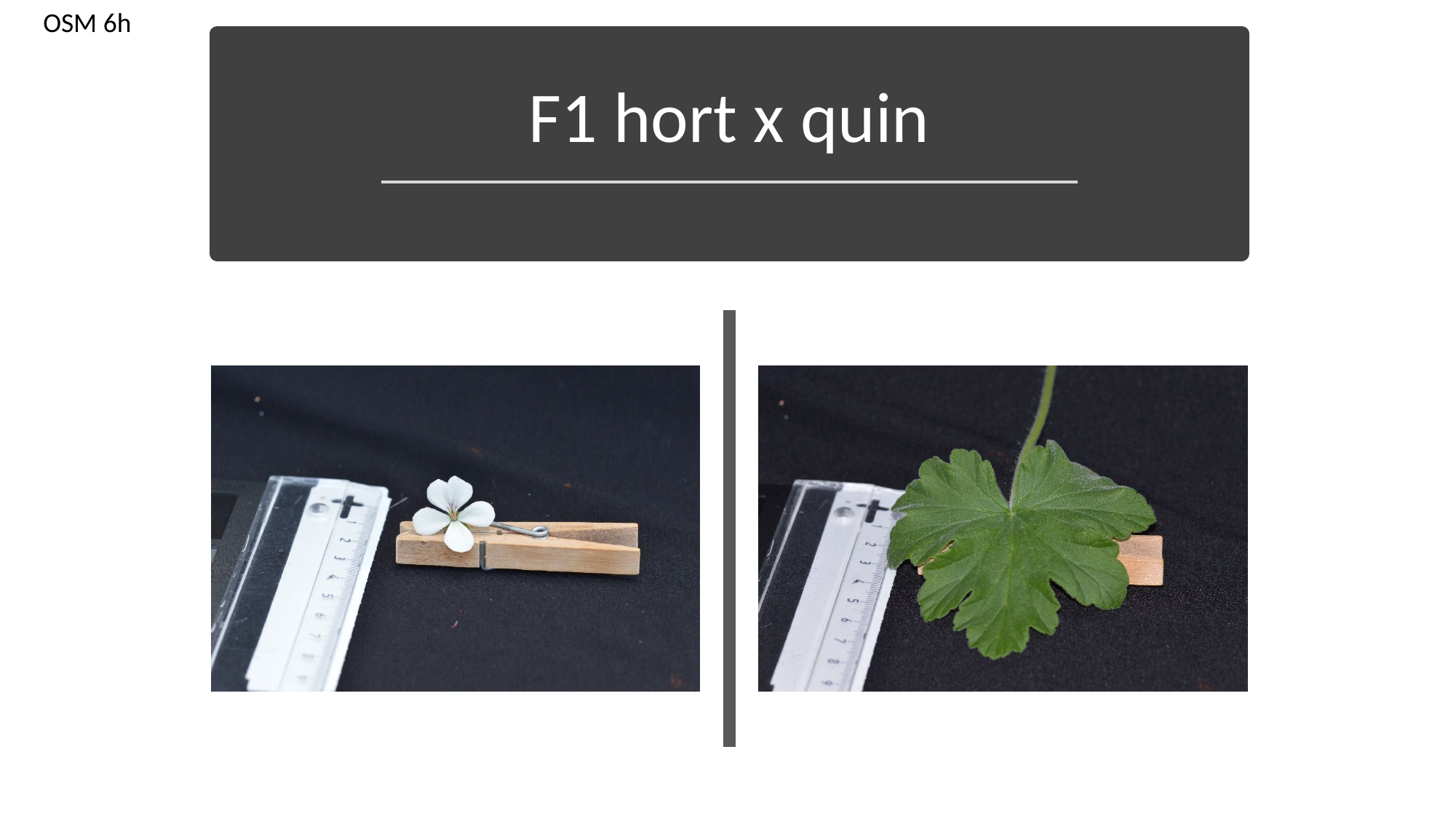

OSM 6h
# F1 hort x quin

## Slide 17
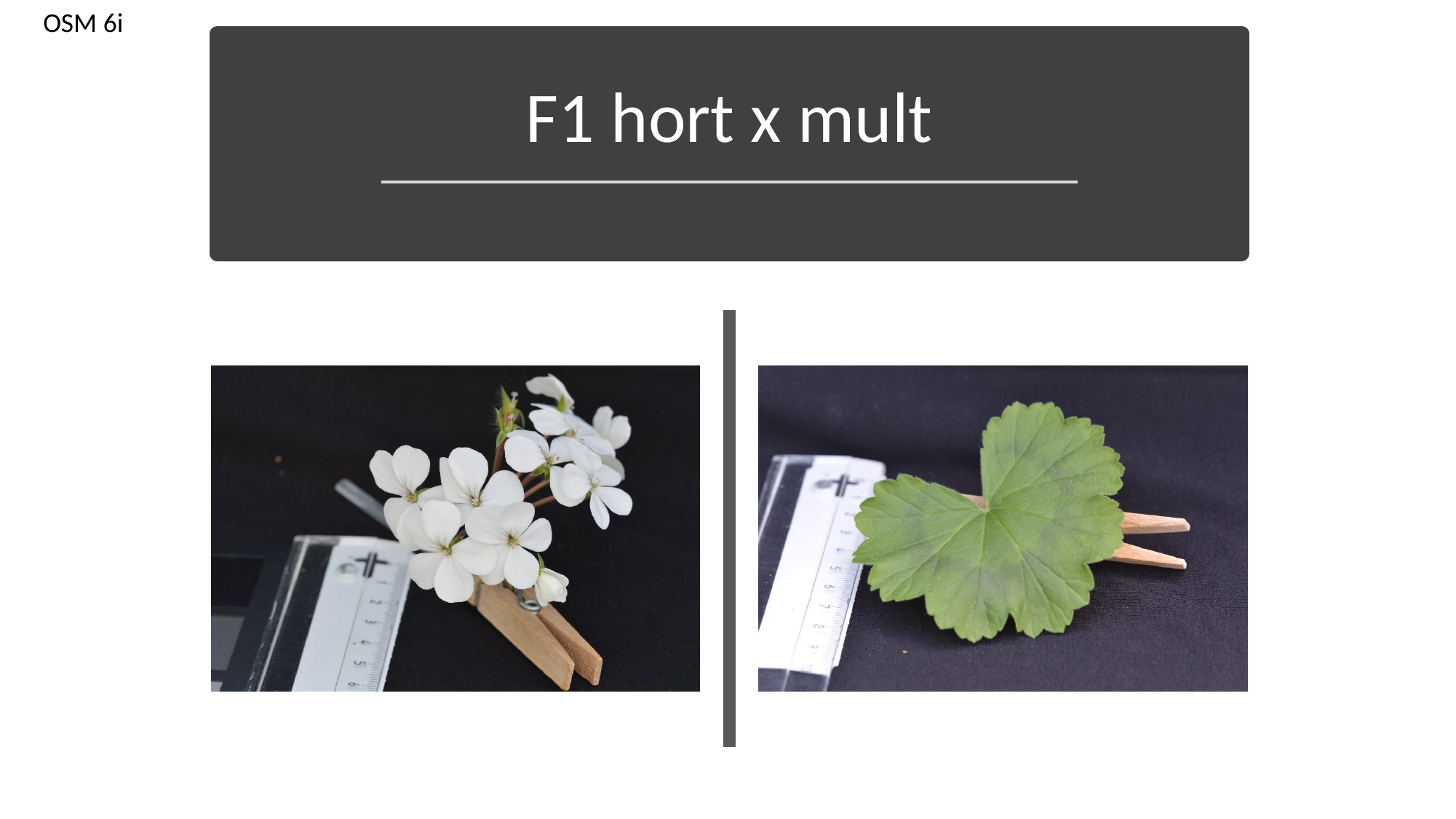

OSM 6i
# F1 hort x mult

## Slide 18
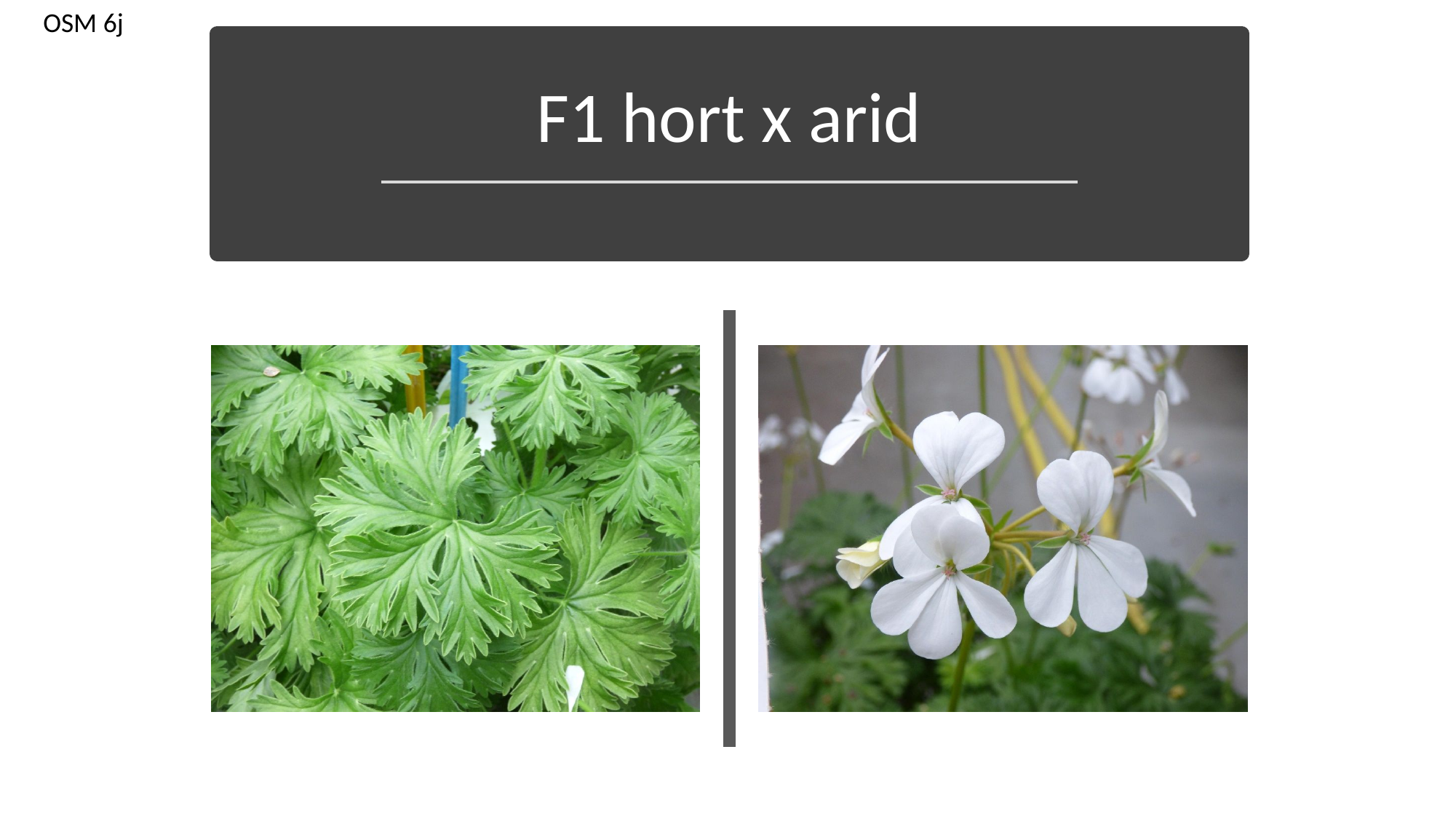

OSM 6j
# F1 hort x arid

## Slide 19
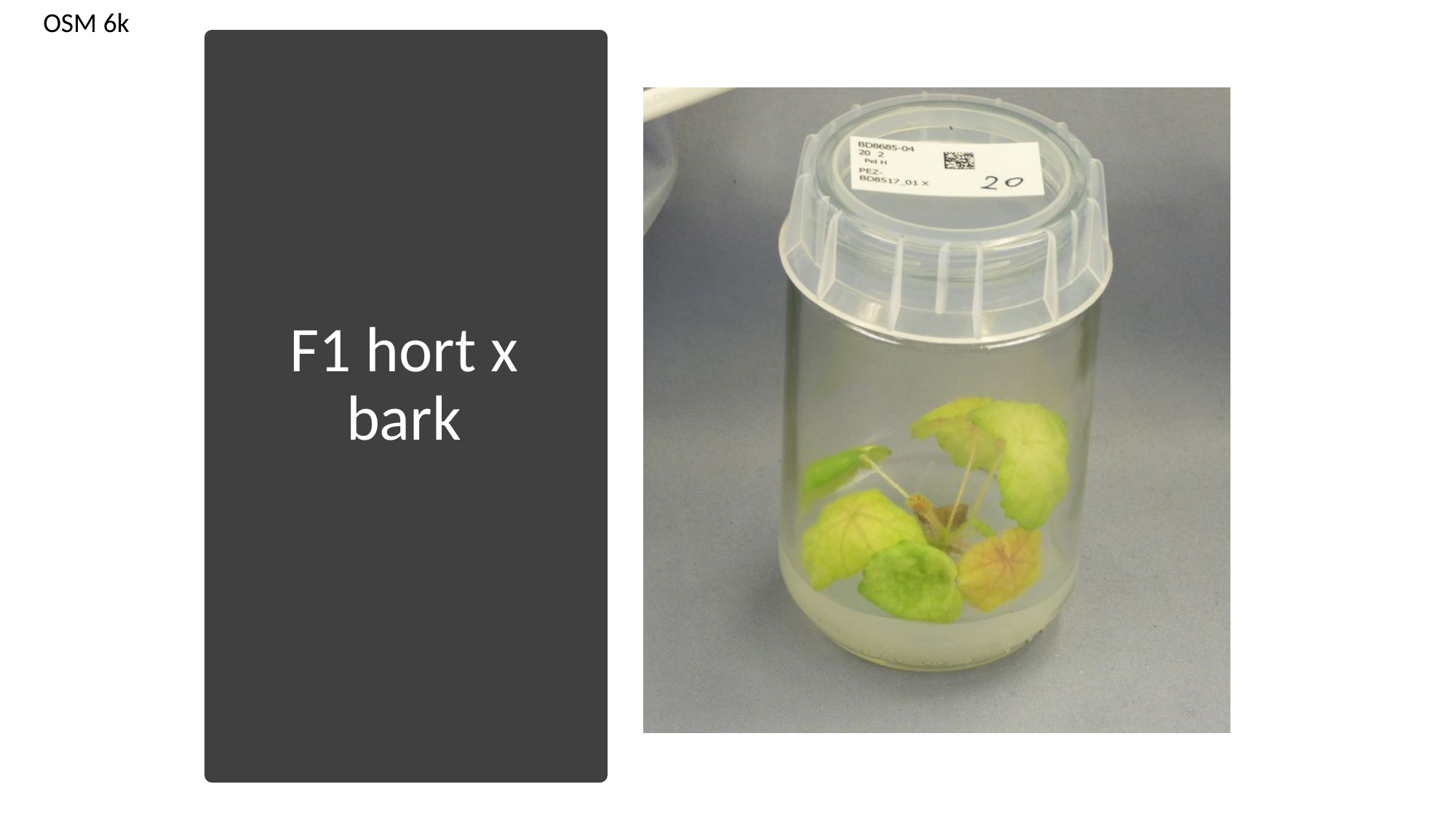

OSM 6k
# F1 hort x bark
